# Supplementary material for: Associations between vision impairment and driving and the effectiveness of vision-related interventions: protocol for a systematic review and meta-analysis
Source: BMJ Open. 2020 Nov 5;10(11):e040881. doi: 10.1136/bmjopen-2020-040881 (PMC7646345; doi:10.1136/bmjopen-2020-040881)
Supplement: Supplementary data [file bmjopen-2020-040881supp002.pdf]

Appendix 2. MEDLINE (OVID) search strategy

1. exp Eye Diseases/
2. exp Cataract Extraction/
3. Lens Implantation, Intraocular/
4. Lenses, Intraocular/
5. cataract$.tw.
6. ((intraocular or intra ocular) adj3 lens$).tw.
7. (IOL or IOLs).tw.
8. Vision Tests/
9. Visual Acuity/
10. exp Refractive Errors/
11. Visual Fields/
12. Visual Field Tests/
13. Contrast Sensitivity/
14. Depth Perception/
15. (visual adj2 (acuit$ or field$)).tw.
16. contrast sensitivity.tw.
17. (depth perception or stereopsis).tw.
18. ((impair$ or decreas$ or declin$) adj3 (vision or visual$ or sight$)).tw.
19. (improv$ adj3 (vision or visual$ or sight$)).tw.
20. ((visual or vision) adj2 function$).tw.
21. exp Vision, Ocular/
22. Vision Screening/
23. or/1-22
24. Mass Screening/
25. ((eye$ or sight or vision or visual$) adj2 (test$ or screen$ or exam$ or diagnos$ or assess$)).tw
26. 24 and 25
27. 23 or 26
28. exp Motor Vehicles/
29. exp Automobile Driving/
30. Accidents, Traffic/
31. (driver$ or driving).tw.
32. (automobile$ or car or cars or vehicle$).tw.
33. (motoring or motorcar or "motor car" or "motor cars").tw.
34. crash$.tw.
35. ((road or traffic) adj2 injur$).tw.
36. ((road or traffic or motor) adj2 (accident$ or incident$)).tw.
37. ((road or traffic or motor) adj2 collision$).tw.
38. or/28-37
39. epidemiologic studies/ or case-control studies/ or cohort studies/ or observational study/ or follow-up studies/ or longitudinal studies/ or prospective studies/ or retrospective studies/ or controlled before-after studies/ or cross-sectional studies/ or historically controlled study/ or interrupted time series analysis/
40. epidemiologic methods/ or focus groups/ or interviews as topic/ or exp "surveys and questionnaires"/
41. epidemiologic research design/ or control groups/ or cross-over studies/ or double-blind method/ or meta-analysis as topic/ or network meta-analysis/ or random allocation/ or single-blind method/
42. epidemiologic methods/ or clinical trials as topic/ or feasibility studies/ or multicenter studies as topic/ or pilot projects/ or sampling studies/ or twin studies as topic/

43. randomized controlled trial/ or controlled clinical trials as topic/ or randomized controlled trials as topic/
44. comparative study/ or evaluation studies/ or meta-analysis/ or review/ or multicenter study/ or "systematic review"/ or validation studies/
45. health surveys/
46. outcome assessment, health care/
47. risk factors/
48. self report/
49. (population or cohort or observation$ or intervention$ or prospective or retrospective or comparative).tw.
50. (questionnaire$ or survey$).tw.
51. (randomized or randomised or randomly or RCT).tw.
52. (systematic review or meta-analysis).tw.
53. (before adj2 after).tw.
54. (case$ adj2 control$).tw.
55. (cross adj1 section$).tw.
56. or/39-55
57. 27 and 38
58. 56 and 57
59. vehicle-controlled.tw.
60. (vehicle adj3 inject$).tw.
61. 59 or 60
62. 58 not 61
63. (animal$ or mouse or mice$ or dog or canine or rat or rats or primate$).ti.
64. (dry eye or cell$ or mutation$ or genes or genome or sequencing).ti.
65. or/63-64
66. 62 not 65
67. limit 66 to english language
68. exp case reports/
69. (case adj2 report$).tw.
70. 68 or 69
71. 67 not 70
72. limit 71 to (editorial or letter)
73. 71 not 72
