# Supplementary material for: Associations between vision impairment and driving and the effectiveness of vision-related interventions: protocol for a systematic review and meta-analysis
Source: BMJ Open. 2020 Nov 5;10(11):e040881. doi: 10.1136/bmjopen-2020-040881 (PMC7646345; doi:10.1136/bmjopen-2020-040881)
Supplement: Supplementary data [file bmjopen-2020-040881supp003.pdf]

Appendix 3. EMBASE Search Strategy

1. exp eye disease/
2. exp cataract extraction/
3. lens implantation/
4. lens implant/
5. cataract$.tw.
6. ((intraocular or intra ocular) adj3 lens$).tw.
7. (IOL or IOLs).tw.
8. vision test/
9. visual acuity/
10. refractive error/
11. visual field/
12. perimetry/
13. contrast sensitivity/
14. depth perception/
15. (visual adj2 (acuit$ or field$)).tw.
16. contrast sensitivity.tw.
17. (depth perception or stereopsis).tw.
18. ((impair$ or decreas$ or declin$) adj3 (vision or visual$ or sight$)).tw.
19. (improv$ adj3 (vision or visual$ or sight$)).tw.
20. ((visual or vision) adj2 function$).tw.
21. vision/
22. or/1-21
23. mass screening/
24. ((eye$ or sight or vision or visual$) adj2 (test$ or screen$ or exam$ or diagnos$ or assess$)).tw.
25. 23 and 24
26. 22 or 25
27. exp car driving/
28. exp motor vehicle/
29. traffic accident/
30. (driver$ or driving).tw.
31. (automobile$ or car or cars or vehicle$).tw.
32. (motoring or motorcar or "motor car" or "motor cars").tw.
33. crash$.tw.
34. ((road or traffic) adj2 injur$).tw.
35. ((road or traffic or motor) adj2 (accident$ or incident$)).tw.
36. ((road or traffic or motor) adj2 collision$).tw.
37. or/27-36
38. study design/
39. controlled clinical trial/
40. case control study/
41. cohort analysis/
42. observational study/
43. follow up/
44. longitudinal study/
45. prospective study/
46. retrospective study/
47. epidemiology/
48. cross-sectional study/
49. control group/

50. crossover procedure/
51. "meta analysis (topic)"/
52. network meta-analysis/
53. randomization/
54. single blind procedure/
55. double blind procedure/
56. "clinical trial (topic)"/
57. "controlled clinical trial (topic)"/
58. "randomized controlled trial (topic)"/
59. "multicenter study (topic)"/
60. feasibility study/
61. pilot study/
62. comparative study/
63. evaluation study/
64. multicenter study/
65. randomized controlled trial/
66. meta analysis/
67. "systematic review"/
68. validation study/
69. interview/
70. questionnaire/
71. outcome assessment/
72. "systematic review (topic)"/
73. health survey/
74. risk factor/
75. self report/
76. evidence based practice/
77. (population or cohort or observation$ or intervention$ or prospective or retrospective or comparative).tw.
78. (questionnaire$ or survey$).tw.
79. (randomized or randomised or randomly or RCT).tw.
80. (systematic review or meta-analysis).tw.
81. (before adj2 after).tw.
82. (case$ adj2 control$).tw.
83. (cross adj1 section$).tw.
84. or/38-83
85. 26 and 37
86. 84 and 85
87. vehicle-controlled.tw.
88. (vehicle adj3 inject$).tw.
89. or/87-88
90. 86 not 89
91. (animal$ or mouse or mice$ or dog or canine or rat or rats or primate$).ti.
92. (dry eye or cell$ or mutation$ or genes or genome or sequencing).ti.
93. or/91-92
94. 90 not 93
95. limit 94 to conference abstract status
96. 94 not 95
97. limit 96 to english language
98. exp case report/
99. (case adj2 report$).tw.

100. or/98-99
101. 97 not 100
102. limit 101 to (conference paper or "conference review" or editorial or letter or note)
103. 101 not 102
