# Supplementary material for: Associations between vision impairment and driving and the effectiveness of vision-related interventions: protocol for a systematic review and meta-analysis
Source: BMJ Open. 2020 Nov 5;10(11):e040881. doi: 10.1136/bmjopen-2020-040881 (PMC7646345; doi:10.1136/bmjopen-2020-040881)
Supplement: Supplementary data [file bmjopen-2020-040881supp004.pdf]

Appendix 4. GLOBAL HEALTH Search Strategy

1. exp eye diseases/
2. exp vision disorders/
3. cataract$.tw.
4. ((intraocular or intra ocular) adj3 lens$).tw.
5. (IOL or IOLs).tw.
6. (visual adj2 (acuit$ or field$)).tw.
7. contrast sensitivity.tw.
8. (depth perception or stereopsis).tw.
9. ((impair$ or decreas$ or declin$) adj3 (vision or visual$ or sight$)).tw.
10. (improv$ adj3 (vision or visual$ or sight$)).tw.
11. ((visual or vision) adj2 function$).tw.
12. ((eye$ or sight or vision or visual$) adj2 (test$ or screen$ or exam$ or diagnos$ or assess$)).tw.
13. or/1-12
14. drivers/
15. vehicles/
16. motor cars/
17. traffic/
18. traffic accidents/
19. (driver$ or driving).tw.
20. (automobile$ or car or cars or vehicle$).tw.
21. (motoring or motorcar or "motor car" or "motor cars").tw.
22. crash$.tw.
23. ((road or traffic) adj2 injur$).tw.
24. ((road or traffic or motor) adj2 (accident$ or incident$)).tw.
25. ((road or traffic or motor) adj2 collision$).tw.
26. or/14-25
27. cohort studies/
28. case-control studies/
29. longitudinal studies/
30. retrospective studies/
31. epidemiology/
32. exp clinical trials/
33. randomized controlled trials/
34. feasibility studies/
35. pilot projects/
36. meta-analysis/
37. systematic reviews/
38. reviews/
39. questionnaires/
40. surveys/
41. epidemiological surveys/
42. risk factors/
43. (population or cohort or observation$ or intervention$ or prospective or retrospective or comparative).tw.
44. (questionnaire$ or survey$).tw.
45. (randomized or randomised or randomly or RCT).tw.
46. (systematic review or meta-analysis).tw.
47. (before adj2 after).tw.
48. (case$ adj2 control$).tw.

49. (cross adj1 section$).tw.
50. or/27-49
51. 13 and 26
52. 50 and 51
53. (animal$ or mouse or mice$ or dog or canine or rat or rats or primate$).ti.
54. (dry eye or cell$ or mutation$ or genes or genome or sequencing).ti.
55. 53 or 54
56. 52 not 55
57. limit 56 to english language
58. case reports/
59. (case adj2 report$).tw.
60. 58 or 59
61. 57 not 60
62. limit 61 to (conference or conference paper or conference proceedings or correspondence or editorial or thesis)
63. 61 not 62
