# Supplementary material for: Associations between vision impairment and driving and the effectiveness of vision-related interventions: protocol for a systematic review and meta-analysis
Source: BMJ Open. 2020 Nov 5;10(11):e040881. doi: 10.1136/bmjopen-2020-040881 (PMC7646345; doi:10.1136/bmjopen-2020-040881)
Supplement: Author's manuscript [file bmjopen-2020-040881.draft_revisions.pdf]

BMJ Open is committed to open peer review. As part of this commitment we make the peer review history of every article we publish publicly available.

When an article is published we post the peer reviewers' comments and the authors' responses online. We also post the versions of the paper that were used during peer review. These are the versions that the peer review comments apply to.

The versions of the paper that follow are the versions that were submitted during the peer review process. They are not the versions of record or the final published versions. They should not be cited or distributed as the published version of this manuscript.

BMJ Open is an open access journal and the full, final, typeset and author-corrected version of record of the manuscript is available on our site with no access controls, subscription charges or pay-per-view fees (http://bmjopen.bmj.com).

If you have any questions on BMJ Open's open peer review process please email info.bmjopen@bmj.com

BMJ Open

Vision impairment and driving: protocol for a systematic review and meta-analysis of associations and the effectiveness of vision-related interventions

Journal:	*BMJ Open*
Manuscript ID	bmjopen-2020-040881
Article Type:	Protocol
Date Submitted by the Author:	25-May-2020
Complete List of Authors:	Nguyen, Helen; University of New South Wales<br>Di Tanna, Gian Luca; George Institute for Global Health,<br>Coxon, Kristy; Western Sydney University, School of Health Sciences<br>Brown, Julie; George Institute for Global Health, Injury<br>Ren, Kerrie; University of New South Wales<br>Ramke, Jacqueline; LSHTM,<br>Burton, Matthew J; London School of Hygiene and Tropical Medicine<br>Gordon, Iris<br>Zhang, Justine; London School of Hygiene and Tropical Medicine Faculty of Infectious and Tropical Diseases, International Centre for Eye Health, Clinical Research Department<br>Furtado, João; Universidade de São Paulo Faculdade de Medicina de Ribeirão Preto, Division of Ophthalmology<br>Mdala, Shaffi; Queen Elizabeth Central Hospital, Opthalmology Department<br>Kitema, Gatera Fiston; University of Rwanda College of Medicine and Health Sciences, Opthalmology Department<br>Keay, Lisa; University of New South Wales, School of Optometry and Vision Science; The George Institute for Global Health
Keywords:	PUBLIC HEALTH, OPHTHALMOLOGY, EPIDEMIOLOGY

SCHOLARONE™
Manuscripts

I, the Submitting Author has the right to grant and does grant on behalf of all authors of the Work (as defined in the below author licence), an exclusive licence and/or a non-exclusive licence for contributions from authors who are: i) UK Crown employees; ii) where BMJ has agreed a CC-BY licence shall apply, and/or iii) in accordance with the terms applicable for US Federal Government officers or employees acting as part of their official duties; on a worldwide, perpetual, irrevocable, royalty-free basis to BMJ Publishing Group Ltd ("BMJ") its licensees and where the relevant Journal is co-owned by BMJ to the co-owners of the Journal, to publish the Work in this journal and any other BMJ products and to exploit all rights, as set out in our licence.

The Submitting Author accepts and understands that any supply made under these terms is made by BMJ to the Submitting Author unless you are acting as an employee on behalf of your employer or a postgraduate student of an affiliated institution which is paying any applicable article publishing charge ("APC") for Open Access articles. Where the Submitting Author wishes to make the Work available on an Open Access basis (and intends to pay the relevant APC), the terms of reuse of such Open Access shall be governed by a Creative Commons licence – details of these licences and which Creative Commons licence will apply to this Work are set out in our licence referred to above.

Other than as permitted in any relevant BMJ Author's Self Archiving Policies, I confirm this Work has not been accepted for publication elsewhere, is not being considered for publication elsewhere and does not duplicate material already published. I confirm all authors consent to publication of this Work and authorise the granting of this licence.

For peer review only

Vision impairment and driving: protocol for a systematic review and meta-analysis of associations and the effectiveness of vision-related interventions

Authors: Helen Nguyen[1], Gian Luca Di Tanna[2], Kristy Coxon[3], Julie Brown[2], Kerrie Ren[1], Jacqueline Ramke[4,5], Matthew J Burton[4,6], Iris Gordon[4], Justine H Zhang[4,7], João M. Furtado[8], Shaffi Mdala[9], Gatera Fiston Kitema[10], Lisa Keay[1,2]

Affiliations:

1 School of Optometry and Vision Science, Faculty of Science, The University of New South Wales, NSW, Australia

2 The George Institute for Global Health, Faculty of Medicine, University of New South Wales, NSW, Australia

3 School of Health Sciences, and the Translational Health Research Institute, Western Sydney University, NSW, Australia

4 International Centre for Eye Health, London School of Hygiene & Tropical Medicine, London, United Kingdom, WC1 E7HT

5 School of Optometry and Vision Science, University of Auckland, Auckland, New Zealand

6 Moorfields Eye Hospital, London, UK

7 Manchester Royal Eye Hospital, Manchester, United Kingdom

8 Division of Ophthalmology, Ribeirão Preto Medical School, University of São Paulo, Ribeirão Preto, São Paulo, Brazil

9 Ophthalmology Department, Queen Elizabeth Central Hospital, Blantyre, Malawi

10 Ophthalmology Department, School of Health Sciences, University of Rwanda, Kigali, Rwanda

Corresponding Author: Lisa Keay

Postal Address: School of Optometry and Vision Science, The University of New South Wales, Sydney UNSW NSW 2052, Australia

Email: l.keay@unsw.edu.au

Keywords: visual impairment, road traffic injuries, motor vehicle crashes, driving cessation, driving errors, driving performance, fatal crash involvement

For peer review only - http://bmjopen.bmj.com/site/about/guidelines.xhtml

ABSTRACT

Introduction: Driving is becoming one of the main modes of transport, however, safe driving requires a combination of visual, cognitive, and physical skills. With population ageing, the number of people living with vision impairment is set to increase in the decades ahead. Vision impairment may negatively impact an individual's ability to safely drive. The association between vision impairment and motor vehicle crash involvement or driving participation has yet to be systematically investigated. Further, the evidence for the effectiveness of vision-related interventions aimed at decreasing crashes and driving errors has not been synthesised.

Methods and Analysis: A search will be conducted for relevant studies on Medline (Ovid), EMBASE, and Global Health from their inception without date or geographical restrictions. Two investigators will independently screen abstracts and full-texts using Covidence software with conflicts resolved by a third investigator. Data extraction will be conducted on all included studies, and their quality assessed to determine risk of bias using the Joanna Briggs Institute (JBI) Critical Appraisal Tools. Outcome measures include crash risk, driving cessation, and surrogate measures of driving safety (e.g. driving errors and performance). The results of this review will be reported using the Preferred Reporting Items for Systematic Review and Meta-Analysis (PRISMA) guideline. Meta-analysis will be undertaken for outcomes with sufficient data and reported following the Meta-analyses of Observational Studies in Epidemiology (MOOSE) guideline. Where statistical pooling is not feasible or appropriate, narrative summaries will be presented following the Synthesis Without Meta-analysis (SWiM) in systematic reviews guideline.

Ethics and dissemination: This review will only report on published data thus no ethics approval is required. Results will be included in the *Lancet Global Health* Commission on Global Eye Health, published in a peer-reviewed journal, and presented at relevant conferences.

PROSPERO registration number: CRD42020172153

For peer review only

For peer review only - http://bmjopen.bmj.com/site/about/guidelines.xhtml

STRENGTHS AND LIMITATIONS OF THIS STUDY

- Results from this review will present the first systematic collection of up-to-date evidence for the influence of vision impairment on road traffic injuries (RTIs) and the corresponding benefits of interventions to restore vision.
- As there are no time or place restrictions in the criteria for included studies this review will capture a large portion of English-language publications on this area
- Publication bias may arise as this review only looks at published studies in English so research from non-English speaking countries or those not yet in an open-access depository will be missed.
- Another potential limitation is that interventions and outcome measures may be highly heterogeneous which will affect the conclusions drawn from the results and prevent meta-analyses to be conducted for select outcomes.

For peer review only

For peer review only - http://bmjopen.bmj.com/site/about/guidelines.xhtml

INTRODUCTION

According to the World Health Organisation (WHO),(1) approximately 1.35 million people die each year from road traffic injuries (RTIs), making it the eighth leading cause of death globally. Low- and middle income countries (LMICs) have lower rates of vehicle ownership compared to high-income countries (HIC), but over 90% of RTI fatalities occur in LMICs with the highest death rates in Africa. Without interventions, disparities in LMICs are set to grow alongside increases in motorisation consistent with globalisation. RTIs make up a major proportion of a country's economic and social burden,(2-4) and account for almost 30% of global injury-related disability.(2) In the face of increasing motorisation, achieving absolute reductions in RTIs is a challenge, especially for vulnerable road users such as pedestrians and users of powered two and three wheeler vehicles. This challenge has a direct impact on the UN's Sustainable Development Goals (SDGs), in particularly Target 3.6 which called for a halving of global road deaths by 2020, and Target 11.2 which called for safe and sustainable transport systems, especially for vulnerable road users.(5)

Motor vehicle crashes (MVCs), and by extension RTIs, however, are preventable. It has long been established that MVCs are multifactorial involving host (human), agent (vehicles and equipment), and environmental (physical and socioeconomic) factors(6) and this has evolved to the Safe System Approach endorsed in the United Nations Road Safety Collaboration's Decade of Action for Road Safety (2011-2020).(7) Road safety programs, such as the *Bloomberg Initiative for Global Road Safety (2015-2019)* focus on improving road safety through legislation in LMICs,(8) thus addressing the environmental and agent risks of RTIs. Beyond road infrastructure and vehicle quality, human driving behaviours or 'human factors' also contribute to RTI rates and are an intrinsic part of the Safe System Approach. Safe driving requires individuals to have a range of physical, visual, and cognitive skills. In addition to specific eye diseases, age-related functional declines across a range of domains, including vision, can reduce confidence in driving ability.(9) Poor visual acuity and contrast sensitivity, visual field loss, and glare sensitivity have all been identified as potential factors contributing to poor driving performance and increased MVCs.(10)

Due to the high visual demands needed to drive safely, standards have been set for vision, mostly for visual acuity. Jurisdictional control is used to identify individuals with vision impairment and restrict their access to driving privileges. However, a systematic review by Dobbs (2008) suggested that licencing policies aimed at identifying at-risk older drivers may not be effective in decreasing crash rates.(11) Conversely, in-person renewal policies which include vision tests completed at licence renewal centres, have been shown to reduce crash rates in older drivers.(12) An American study analysing data from the National Highway Traffic Safety Administration Fatal Accident Reporting

For peer review only - http://bmjopen.bmj.com/site/about/guidelines.xhtml

System found drivers aged 70 years and older who underwent visual acuity examinations during their licence renewals had lower fatal crash risks than their non-vision tested peers (RR 0.93; 95% CI 0.89 – 0.97).(13) However, the literature remains divided in its support for using visual acuity alone as a predictor of MVC involvement and high-risk driving behaviours.(14) A Cochrane review, updated twice, examined the benefits of different vision screening procedures in randomised-controlled trials (RCTs) aimed at preventing RTIs and fatalities in older drivers.(15, 16) Unfortunately, no RCTs met the inclusion criteria for the review at the time these reviews were conducted.

There is substantial literature investigating how vision impairment, and other aspects of function, affect road safety. Measures of driving safety have included indirect measures such as performance on driving simulators, on-road driving assessments, naturalistic driving or in-vehicle monitoring as well as direct measures of RTI and MVC rates from self-report or administrative datasets.(17, 18) However, the evidence for the influence of vision loss on MVCs and the corresponding benefits of interventions to restore vision have not been systematically evaluated. Since older drivers have higher crash involvement(19) and greater prevalence of eye diseases,(20) most research investigate older drivers and their risks of crashes and injuries. However, it is important to document the impact of vision impairment across all age groups. Further, information is also needed about specific eye diseases and types of vision impairment to inform interventions to screen for poor vision in drivers, and interventions to rehabilitate vision, thereby enhancing driver safety and continued ability to drive.

The aim of this systematic review is to: 1) describe the associations between vision impairment and risk of road crash involvement across the lifespan, and 2) evaluate vision-related interventions to reduce crash risk. Secondary outcomes are driving cessation and surrogate measures of crash risk such as on-road driving errors.

METHODS AND ANALYSIS

This systematic review protocol was drafted using the International Prospective Register of Systematic Reviews (PROSPERO) as a guideline and registered in PROSPERO (28/04/2020; CRD42020172153 [https://www.crd.york.ac.uk/prospero/display_record.php?ID=CRD42020172153]). Any changes to the protocol will be updated in PROSPERO. The protocol is prepared in accordance to the Preferred Reporting Items for Systematic review and Meta-Analysis Protocols (PRISMA-P) statement (Appendix no. 1).(21)

Eligibility Criteria

For peer review only - http://bmjopen.bmj.com/site/about/guidelines.xhtml

This review will include human studies in the English language with full-text available. Both interventional (randomised controlled trials and quasi-experimental) and observational (cohort, cross-sectional, and case-control) studies will be considered. Systematic reviews will be included if meta-analysis was performed. For systematic reviews without meta-analysis, the reference list will be examined for potentially relevant articles, but the systematic review itself will not be included. All literature reviews, commentary articles, dissertations, abstracts, editorials, and conference presentations will be excluded. Studies which used driving simulators will be excluded as these are laboratory studies with only indirect measures of driving performance. Real-life driving experiences, such as limited exposure to driving at night, in bad weather, or during rush hour, may not be reflected in a simulation.(22) Additionally, the validity of driving simulator results are highly dependent upon the type of simulation program used and what kind of driving manoeuvre is being investigated.(23) Studies investigating either self-regulatory behaviours, such as night driving avoidance and decreasing travel mileage, or self-reported measures of driving safety, will also be excluded.

The population of focus will be drivers of four-wheeled motorised vehicles such as cars, buses, and trucks. Studies including drivers who have specific medical conditions (e.g. dementia, epilepsy, and stroke), or vision difficulties due to other medical factors (e.g. hemianopia caused by brain damage) will not be included. Similarly, articles where vision status is not reported will be excluded.

Exposures in the included studies will encompass all types of vision impairment including visual acuity, contrast sensitivity, visual field loss as well as impairments associated with specific eye diseases including but not limited to glaucoma, cataracts, aged-related macular degeneration (AMD), diabetic retinopathy (DR), stereopsis disorders, and colour vision deficiencies. Vision impairments can be categorised by the specific eye diseases or by specific measures of vision which can negatively impact normal everyday functioning. Interventions can include vision screening, refractive correction, cataract surgery or other measures to restore and improve vision of drivers in order to maintain driving participation, promote safe driving and reduce risk of crash involvement. The exposure comparators of included studies will be drivers who either do not have a vision impairment or have not received a vision-related intervention, within a timeframe chosen by the study in question.

Outcome Measures

The primary outcome measure is MVC involvement including fatal MVC involvement. Data on crash involvement and its severity can either come from self-reported surveys or data linkage with

For peer review only

government and/or hospital records. Data from self-reported surveys will ensure that MVCs which were not serious enough to warrant a police or hospital report will be also be included.

Driving cessation and surrogate measures of driving safety will be the secondary outcomes. The surrogate measures of driving safety can include scores of driving performance from on-road driving tests or 'naturalistic' in-vehicle monitoring looking at manoeuvres such as lane keeping, braking, and abidance of road signage like traffic lights, stop and give way signs. A pass/fail threshold for driving performance scores will be decided upon by all investigators.

Search Strategy

Electronic database search will be conducted by the Cochrane Eyes and Vision Information Specialist (IG) on Medline (Ovid), EMBASE, and Global Health from their inception to March 2020. Appendices 2, 3, and 4 shows the search strategies for Medline, EMBASE, and Global Health, respectively. Additional potentially relevant studies will be sought by experts in the field by checking the reference lists of included studies, and checking the reference list of narrative systematic reviews identified in the search.

Data Collection and Analysis

Data Management and Selection

Each title and abstracts will be screened by two investigators independently (from HN, KR, JR, JZ, SM, JF, GFK) using Covidence systematic review management software (Veritas Health Innovation, Melbourne, Australia; available at https://www.covidence.org/home). Full-text review of potentially relevant articles will then be conducted by two investigators independently. Discrepancies will be discussed and resolved via consultation with a third investigator.

Data Extraction

Data extraction will be completed independently by two investigators (from among the same seven investigators). Data from included studies will be extracted using adaptions of the Joanna Briggs Institute (JBI) template for systematic reviews and observational studies (including cohort, cross-sectional, and case-control studies).(24) Adapted Cochrane templates will be used to extract data from randomized controlled trials and quasi-experimental studies.(25)

Quality Assessment

A quality assessment to determine an overall risk of bias will be carried out on all included studies independently by two investigators (from the seven investigators mentioned previously). Conflicts will be resolved by a third investigator. Relevant JBI critical appraisal tools will be used to evaluate

For peer review only - http://bmjopen.bmj.com/site/about/guidelines.xhtml

randomised controlled trials, quasi-experimental studies, systematic reviews, cohort studies, cross-sectional studies, and case-control studies.(26)

Data Synthesis Strategy

Measures of association between vision impairment/vision related interventions and MVC involvement, driving cessation and surrogate measures of driving safety will be summarized according to the outcome measures reported in the primary studies. In particular, appropriate hazard ratio (HR), risk ratio (RR), and odds ratio (OR) for binary data and (standardized) mean differences for continuous data will be statistically pooled. When the same outcome is reported as dichotomous data in some studies and as continuous data in others, these studies will be pooled by expressing the ORs as standardized mean differences and vice versa.(27) P-values of the driving outcomes will also be reported where appropriate.

Where it is not possible or suitable to statistically pool the studies, a narrative summary of the findings will be used instead. Narrative summaries will follow the Synthesis Without Meta-analysis (SWiM) reporting guidelines.(28) Heterogeneity for all included studies will be assessed clinically, methodologically and statistically. Clinical heterogeneity will be accessed by comparing the differences between the participant characteristics (e.g. age, sex, eye disease), interventions and outcomes measured. The design and quality of included studies will be compared to access methodological heterogeneity. Statistical heterogeneity will be explored by formal statistical test of heterogeneity, subgroup analyses and, if feasible, by meta-regression. Inconsistency of the effect sizes across the studies will be assessed by the proportion of variability in the effect sizes of the included studies due to heterogeneity (and not by sampling error) using I^2. Estimates will be pooled using random effects models with fixed effect models results also reported regardless of the values of I^2, and prediction intervals to allow for expected effects of future studies to be extrapolated based upon the current evidence.(29)

The following outcomes will be assessed using meta-analysis where possible: crash involvement, driving cessation, and surrogate measures of un-safe driving i.e. driving errors and driving performance. Furthermore meta-analyses for each of the different eye diseases, and studies from LMIC settings will also be performed independently for each outcome of interest if possible. The Meta-analysis of Observational Studies in Epidemiology (MOOSE) guidelines will be used to guide reporting.(30) The Grading of Recommendations Assessment, Development and Evaluation (GRADE) approach will be used to assess the quality of evidence in the meta-analyses.(31)

Sensitivity analysis will be performed on low risk of bias studies whilst the meta-analysis will include all studies. This will assist with verifying the strength of the study findings and to assess how

For peer review only - http://bmjopen.bmj.com/site/about/guidelines.xhtml

different methodologies, sample sizes and statistical analyses have affected this study's results. Furthermore, funnel plots will be used to assess publication bias.

Corresponding authors from publications dated 2010 onwards with missing data of potential use will be approached via email, up to a maximum of 3 attempts, to request further information. Any unobtainable data will be noted alongside all attempts to contact the respective authors. Even though only available data will be used for the meta-analysis, the effects of any missing data will be considered and their effects discussed in the overall final review.

Patient and Public Involvement

This review will only be looking at existing published literature. No patient or public involvement is currently planned for the design and execution of this review, however public participation may be sought for this review's dissemination.

ETHICS AND DISSEMINATION

As this review will only be focusing on currently published literature, ethics approval is not required. Results from this systematic review will be published in an open peer-reviewed journal and will form part of the ongoing *Lancet Global Health* Commission on Global Eye Health.(32) Where relevant, it will also be presented at conferences.

DISCUSSION

Significance of this review

The findings of this systematic review may influence future road safety policies on driving and care for people who would like to drive but have eye conditions which can cause vision impairment. By identifying the associations between vision and crash involvement, vision-related screening tests for licencing may be reconsidered and updated to increase relevance to driving safety. As mentioned previously, most reviews on driving with vision impairment have been limited to older drivers and the effects of different licencing renewal procedures on their ability to drive. Even though older drivers are at higher risk,(20) this review will seek to capture data on driving and vision impairment for all age groups.

The eligibility criteria for included studies for this review will ensure that global data on vision and driving will be captured. Currently, MVC-related societal burdens and injury- related disability burdens in LMICs are poorly understood which may partially explain why cost-effective interventions in these countries are rarely undertaken.(2) LMICs tend to focus on legislative interventions, followed by education/training workshops, public awareness campaigns, enforcement measures, speed control and infrastructure improvements.(33) Current data on human factors specifically

For peer review only - http://bmjopen.bmj.com/site/about/guidelines.xhtml

For peer review only

related to vision impairment in LMICs reported in this review may inform future evidence-based policies on licencing and/or screening policies to address these gaps.

This review may also be advantageous for the development of future vision-related interventions aimed at improving driving outcomes, in particular information on which interventions work best and for which eye disease. This in turn will further strengthen the evidence needed to improve policies around road safety for individuals with vision impairments.

For peer review only

For peer review only - http://bmjopen.bmj.com/site/about/guidelines.xhtml

REFERENCES

1 World Health Organisation. Road Traffic Injuries 2020; https://www.who.int/news-room/fact-sheets/detail/road-traffic-injuries (accessed April 2020)

2 Bachani AM, Peden M, Gururaj G, et al. Road Traffic Injuries. In: Mock CN, Nugent R, Kobusingye O, Smith KR, eds. Injury Prevention and Environmental Health. Washington (DC): The International Bank for Reconstruction and Development / The World Bank © 2017 International Bank for Reconstruction and Development / The World Bank.; 2017.

3 Chen S, Kuhn M, Prettner K, et al. The global macroeconomic burden of road injuries: estimates and projections for 166 countries. *Lancet Planet Health* 2019;3(9):e390-e8. doi: 10.1016/S2542-5196(19)30170-6

4 Wijnen W, Stipdonk H. Social costs of road crashes: An international analysis. *Accid Anal Prev* 2016;94:97-106. doi: 10.1016/j.aap.2016.05.005 [published Online First: 3 June 2016]

5 United Nations. #Envision2030: 17 goals to transform the world for persons with disabilities; https://www.un.org/development/desa/disabilities/envision2030.html (accessed April 2020)

6 Haddon W, Jr. Energy damage and the ten countermeasure strategies. *J Trauma* 1973;13(4):321-31. doi: 10.1177/001872087301500407

7 The Department of Infrastructure, Regional Development and Communications: Australian Government. Decade of Action for Road Safety 2011-2020 2014; https://www.infrastructure.gov.au/roads/safety/decade_of_action/index.aspx (accessed April 2020)

8 Global Road Safety Partnership. Bloomberg Initiative for Global Road Safety; https://www.grsproadsafety.org/programmes/bloomberg-initiative-global-road-safety/ (accessed April 2020)

9 Miller SM, Taylor-Piliae RE, Insel KC. The association of physical activity, cognitive processes and automobile driving ability in older adults: A review of the literature. *Geriatr Nurs* 2016;37(4):313-20. doi: 10.1016/j.gerinurse.2016.05.004 [published Online First: 31 May 2016]

10 Rubin GS, Ng ES, Bandeen-Roche K, et al. A prospective, population-based study of the role of visual impairment in motor vehicle crashes among older drivers: the SEE study. *Invest Ophthalmol Vis Sci* 2007;48(4):1483-91. doi: 10.1167/iovs.06-0474

11 Dobbs BM. Aging baby boomers--a blessing or challenge for driver licensing authorities. *Traffic Inj Prev* 2008;9(4):379-86. doi: 10.1080/15389580802045823

12 Grabowski DC, Campbell CM, Morrisey MA. Elderly licensure laws and motor vehicle fatalities. *JAMA* 2004;291(23):2840-6. doi: 10.1001/jama.291.23.2840

13 Levy DT, Vernick JS, Howard KA. Relationship between driver's license renewal policies and fatal crashes involving drivers 70 years or older. *JAMA* 1995;274(13):1026-30. doi: 0.1001/jama.1995.03530130032024

14 Owsley C, McGwin G, Jr. Vision impairment and driving. *Surv Ophthalmol* 1999;43(6):535-50.

For peer review only - http://bmjopen.bmj.com/site/about/guidelines.xhtml

15 Desapriya E, Harjee R, Brubacher J, et al. Vision screening of older drivers for preventing road traffic injuries and fatalities. *Cochrane Database Syst Rev* 2014(2):Cd006252. doi: 10.1002/14651858.CD006252.pub3

16 Subzwari S, Desapriya E, Babul-Wellar S, et al. Vision screening of older drivers for preventing road traffic injuries and fatalities. *Cochrane Database Syst Rev* 2009(1):Cd006252. doi: 10.1002/14651858.CD006252.pub2

17 Imprialou M, Quddus M. Crash data quality for road safety research: Current state and future directions. *Accid Anal Prev* 2019;130:84-90. doi: 10.1016/j.aap.2017.02.022

18 Kaye SA, Lewis I, Freeman J. Comparison of self-report and objective measures of driving behavior and road safety: A systematic review. *J Safety Res* 2018;65:141-51. doi: 10.1016/j.jsr.2018.02.012

19 Owsley C, Ball K, Sloane ME, et al. Visual/cognitive correlates of vehicle accidents in older drivers. *Psychol Aging* 1991;6(3):403-15. doi: 10.1037//0882-7974.6.3.403

20 Wood JM, Black AA. Ocular disease and driving. *Clin Exp Optom*. 2016;99(5):395-401. doi: 10.1111/cxo.12391 [published Online First: 7 May 2016]

21 Shamseer L, Moher D, Clarke M, et al. Preferred reporting items for systematic review and meta-analysis protocols (PRISMA-P) 2015: elaboration and explanation. *BMJ* 2015;350:g7647. doi: 10.1136/bmj.g7647

22 Keay L, West SK. Vision and Driving Performance in Elderly. In: Scholl HPN, Massof RW, West SK eds. Ophthalmology and the Ageing Society. Berlin Heidelberg: Springer-Verlag 2013:193-207.

23 Shechtman O, Classen S, Awadzi K, et al. Comparison of driving errors between on-the-road and simulated driving assessment: a validation study. *Traffic Inj Prev* 2009;10(4):379-85. doi: 10.1080/15389580902894989

24 The Joanna Briggs Institute. Joanna Briggs Institute Reviewer's Manual. 2017. Available from: https://reviewersmanual.joannabriggs.org/ (accessed April 2020)

25 Cochrane: Developmental PaLP. Data Extraction Forms 2014 [Available from: https://dplp.cochrane.org/data-extraction-forms (accessed April 2020).

26 University of Adelaide. Critical Appraisal Tools; https://joannabriggs.org/ebp/critical_appraisal_tools (assessed April 2020)

27 Anzures-Cabrera J, Sarpatwari A, Higgins JP. Expressing findings from meta-analyses of continuous outcomes in terms of risks. *Stat Med* 2011;30(25):2967-85. doi: 10.1002/sim.4298 [published Online First: 8 August 2011]

28 Campbell M, McKenzie JE, Sowden A, et al. Synthesis without meta-analysis (SWiM) in systematic reviews: reporting guideline. *BMJ* 2020;368:l6890. doi: 10.1136/bmj.l6890

29 IntHout J, Ioannidis JP, Rovers MM, et al. Plea for routinely presenting prediction intervals in meta-analysis. *BMJ Open* 2016;6(7):e010247. doi: 10.1136/bmjopen-2015-010247

For peer review only - http://bmjopen.bmj.com/site/about/guidelines.xhtml

30 Stroup DF, Berlin JA, Morton SC, et al. Meta-analysis of observational studies in epidemiology: a proposal for reporting. Meta-analysis Of Observational Studies in Epidemiology (MOOSE) group. *JAMA* 2000;283(15):2008-12. doi: 10.1001/jama.283.15.2008

31 Iorio A, Spencer FA, Falavigna M, et al. Use of GRADE for assessment of evidence about prognosis: rating confidence in estimates of event rates in broad categories of patients. *BMJ* 2015;350:h870. doi: 10.1136/bmj.h870

32 Burton MJ, Faal HB, Ramke J, et al. Announcing The Lancet Global Health Commission on Global Eye Health. *Lancet Glob Health* 2019;7(12):e1612-e3. doi: 10.1016/S2214-109X(19)30450-4 [published Online First: 9 October 2019]

33 Staton C, Vissoci J, Gong E, et al. Road Traffic Injury Prevention Initiatives: A Systematic Review and Metasummary of Effectiveness in Low and Middle Income Countries. *PloS One* 2016;11(1):e0144971. doi: 10.1371/journal.pone.0144971

For peer review only

For peer review only - http://bmjopen.bmj.com/site/about/guidelines.xhtml

Authors' Contributions: HN, LK, and JR conceived the idea for the review. HN drafted and revised the protocol with suggestions from LK, JR, JB, KC, GLdT, MJB, JZ, IG, JFM, SM, and GFK who reviewed the protocol and provided feedback on the draft. IG constructed the search. The final version of the manuscript was approved by all authors.

Funding Statement: MJB is supported by the Wellcome Trust (207472/Z/17/Z). JR is a Commonwealth Rutherford Fellow, funded by the UK government through the Commonwealth Scholarship Commission in the UK. The Lancet Global Health Commission on Global Eye Health is supported by The Queen Elizabeth Diamond Jubilee Trust, The Wellcome Trust, Sightsavers, The Fred Hollows Foundation, The SEVA Foundation, The British Council for the Prevention of Blindness and Christian Blind Mission.

Acknowledgements: None.

Competing Interests: None declared.

Number of Tables: 0

Number of Figures: 0

Data Sharing Statement: Data generated from this review will be available upon reasonable request from HN.

Word Count: 2848

For peer review only

Appendix 1. Preferred Reporting Items for Systematic review and Meta-analysis Protocols (PRISMA-P) 2015 Checklist

Section and topic	Item No	Checklist item	Page Number
**ADMINISTRATIVE INFORMATION**			
Title:			
Identification	1a	Identify the report as a protocol of a systematic review	1
Update	1b	If the protocol is for an update of a previous systematic review, identify as such	N/A
Registration	2	If registered, provide the name of the registry (such as PROSPERO) and registration number	2, 5
Authors:			
Contact	3a	Provide name, institutional affiliation, e-mail address of all protocol authors; provide physical mailing address of corresponding author	1
Contributions	3b	Describe contributions of protocol authors and identify the guarantor of the review	6, 7, 14
Amendments	4	If the protocol represents an amendment of a previously completed or published protocol, identify as such and list changes; otherwise, state plan for documenting important protocol amendments	N/A
Support:			
Sources	5a	Indicate sources of financial or other support for the review	14
Sponsor	5b	Provide name for the review funder and/or sponsor	14
Role of sponsor or funder	5c	Describe roles of funder(s), sponsor(s), and/or institution(s), if any, in developing the protocol	14
**INTRODUCTION**			
Rationale	6	Describe the rationale for the review in the context of what is already known	4, 5
Objectives	7	Provide an explicit statement of the question(s) the review will address with reference to participants, interventions, comparators, and outcomes (PICO)	5
**METHODS**			
Eligibility criteria	8	Specify the study characteristics (such as PICO, study design, setting, time frame) and report characteristics (such as years considered, language, publication status) to be used as criteria for eligibility for the review	5, 6
Information sources	9	Describe all intended information sources (such as electronic databases, contact with study authors, trial registers or other grey literature sources) with planned dates of coverage	6, 7

For peer review only

Search strategy	10	Present draft of search strategy to be used for at least one electronic database, including planned limits, such that it could be repeated	Appendix no. 2-4
Study records:			
Data management	11a	Describe the mechanism(s) that will be used to manage records and data throughout the review	7
Selection process	11b	State the process that will be used for selecting studies (such as two independent reviewers) through each phase of the review (that is, screening, eligibility and inclusion in meta-analysis)	7
Data collection process	11c	Describe planned method of extracting data from reports (such as piloting forms, done independently, in duplicate), any processes for obtaining and confirming data from investigators	7
Data items	12	List and define all variables for which data will be sought (such as PICO items, funding sources), any pre-planned data assumptions and simplifications	6, 7
Outcomes and prioritization	13	List and define all outcomes for which data will be sought, including prioritization of main and additional outcomes, with rationale	6, 7
Risk of bias in individual studies	14	Describe anticipated methods for assessing risk of bias of individual studies, including whether this will be done at the outcome or study level, or both; state how this information will be used in data synthesis	8, 9
Data synthesis	15a	Describe criteria under which study data will be quantitatively synthesised	8
	15b	If data are appropriate for quantitative synthesis, describe planned summary measures, methods of handling data and methods of combining data from studies, including any planned exploration of consistency (such as I^2, Kendall's τ)	8
	15c	Describe any proposed additional analyses (such as sensitivity or subgroup analyses, meta-regression)	8, 9
	15d	If quantitative synthesis is not appropriate, describe the type of summary planned	2, 8
Meta-bias(es)	16	Specify any planned assessment of meta-bias(es) (such as publication bias across studies, selective reporting within studies)	8, 9
Confidence in cumulative evidence	17	Describe how the strength of the body of evidence will be assessed (such as GRADE)	8

For peer review only

Appendix 2. MEDLINE (OVID) search strategy

1. exp Eye Diseases/
2. exp Cataract Extraction/
3. Lens Implantation, Intraocular/
4. Lenses, Intraocular/
5. cataract$.tw.
6. ((intraocular or intra ocular) adj3 lens$).tw.
7. (IOL or IOLs).tw.
8. Vision Tests/
9. Visual Acuity/
10. exp Refractive Errors/
11. Visual Fields/
12. Visual Field Tests/
13. Contrast Sensitivity/
14. Depth Perception/
15. (visual adj2 (acuit$ or field$)).tw.
16. contrast sensitivity.tw.
17. (depth perception or stereopsis).tw.
18. ((impair$ or decreas$ or declin$) adj3 (vision or visual$ or sight$)).tw.
19. (improv$ adj3 (vision or visual$ or sight$)).tw.
20. ((visual or vision) adj2 function$).tw.
21. exp Vision, Ocular/
22. Vision Screening/
23. or/1-22
24. Mass Screening/
25. ((eye$ or sight or vision or visual$) adj2 (test$ or screen$ or exam$ or diagnos$ or assess$)).tw
26. 24 and 25
27. 23 or 26
28. exp Motor Vehicles/
29. exp Automobile Driving/
30. Accidents, Traffic/
31. (driver$ or driving).tw.
32. (automobile$ or car or cars or vehicle$).tw.
33. (motoring or motorcar or "motor car" or "motor cars").tw.
34. crash$.tw.
35. ((road or traffic) adj2 injur$).tw.
36. ((road or traffic or motor) adj2 (accident$ or incident$)).tw.
37. ((road or traffic or motor) adj2 collision$).tw.
38. or/28-37
39. epidemiologic studies/ or case-control studies/ or cohort studies/ or observational study/ or follow-up studies/ or longitudinal studies/ or prospective studies/ or retrospective studies/ or controlled before-after studies/ or cross-sectional studies/ or historically controlled study/ or interrupted time series analysis/
40. epidemiologic methods/ or focus groups/ or interviews as topic/ or exp "surveys and questionnaires"/
41. epidemiologic research design/ or control groups/ or cross-over studies/ or double-blind method/ or meta-analysis as topic/ or network meta-analysis/ or random allocation/ or single-blind method/
42. epidemiologic methods/ or clinical trials as topic/ or feasibility studies/ or multicenter studies as topic/ or pilot projects/ or sampling studies/ or twin studies as topic/

For peer review only

43. randomized controlled trial/ or controlled clinical trials as topic/ or randomized controlled trials as topic/
44. comparative study/ or evaluation studies/ or meta-analysis/ or review/ or multicenter study/ or "systematic review"/ or validation studies/
45. health surveys/
46. outcome assessment, health care/
47. risk factors/
48. self report/
49. (population or cohort or observation$ or intervention$ or prospective or retrospective or comparative).tw.
50. (questionnaire$ or survey$).tw.
51. (randomized or randomised or randomly or RCT).tw.
52. (systematic review or meta-analysis).tw.
53. (before adj2 after).tw.
54. (case$ adj2 control$).tw.
55. (cross adj1 section$).tw.
56. or/39-55
57. 27 and 38
58. 56 and 57
59. vehicle-controlled.tw.
60. (vehicle adj3 inject$).tw.
61. 59 or 60
62. 58 not 61
63. (animal$ or mouse or mice$ or dog or canine or rat or rats or primate$).ti.
64. (dry eye or cell$ or mutation$ or genes or genome or sequencing).ti.
65. or/63-64
66. 62 not 65
67. limit 66 to english language
68. exp case reports/
69. (case adj2 report$).tw.
70. 68 or 69
71. 67 not 70
72. limit 71 to (editorial or letter)
73. 71 not 72

For peer review only

Appendix 3. EMBASE Search Strategy

1. exp eye disease/
2. exp cataract extraction/
3. lens implantation/
4. lens implant/
5. cataract$.tw.
6. ((intraocular or intra ocular) adj3 lens$).tw.
7. (IOL or IOLs).tw.
8. vision test/
9. visual acuity/
10. refractive error/
11. visual field/
12. perimetry/
13. contrast sensitivity/
14. depth perception/
15. (visual adj2 (acuit$ or field$)).tw.
16. contrast sensitivity.tw.
17. (depth perception or stereopsis).tw.
18. ((impair$ or decreas$ or declin$) adj3 (vision or visual$ or sight$)).tw.
19. (improv$ adj3 (vision or visual$ or sight$)).tw.
20. ((visual or vision) adj2 function$).tw.
21. vision/
22. or/1-21
23. mass screening/
24. ((eye$ or sight or vision or visual$) adj2 (test$ or screen$ or exam$ or diagnos$ or assess$)).tw.
25. 23 and 24
26. 22 or 25
27. exp car driving/
28. exp motor vehicle/
29. traffic accident/
30. (driver$ or driving).tw.
31. (automobile$ or car or cars or vehicle$).tw.
32. (motoring or motorcar or "motor car" or "motor cars").tw.
33. crash$.tw.
34. ((road or traffic) adj2 injur$).tw.
35. ((road or traffic or motor) adj2 (accident$ or incident$)).tw.
36. ((road or traffic or motor) adj2 collision$).tw.
37. or/27-36
38. study design/
39. controlled clinical trial/
40. case control study/
41. cohort analysis/
42. observational study/
43. follow up/
44. longitudinal study/
45. prospective study/
46. retrospective study/
47. epidemiology/
48. cross-sectional study/
49. control group/

For peer review only

50. crossover procedure/
51. "meta analysis (topic)"/
52. network meta-analysis/
53. randomization/
54. single blind procedure/
55. double blind procedure/
56. "clinical trial (topic)"/
57. "controlled clinical trial (topic)"/
58. "randomized controlled trial (topic)"/
59. "multicenter study (topic)"/
60. feasibility study/
61. pilot study/
62. comparative study/
63. evaluation study/
64. multicenter study/
65. randomized controlled trial/
66. meta analysis/
67. "systematic review"/
68. validation study/
69. interview/
70. questionnaire/
71. outcome assessment/
72. "systematic review (topic)"/
73. health survey/
74. risk factor/
75. self report/
76. evidence based practice/
77. (population or cohort or observation$ or intervention$ or prospective or retrospective or comparative).tw.
78. (questionnaire$ or survey$).tw.
79. (randomized or randomised or randomly or RCT).tw.
80. (systematic review or meta-analysis).tw.
81. (before adj2 after).tw.
82. (case$ adj2 control$).tw.
83. (cross adj1 section$).tw.
84. or/38-83
85. 26 and 37
86. 84 and 85
87. vehicle-controlled.tw.
88. (vehicle adj3 inject$).tw.
89. or/87-88
90. 86 not 89
91. (animal$ or mouse or mice$ or dog or canine or rat or rats or primate$).ti.
92. (dry eye or cell$ or mutation$ or genes or genome or sequencing).ti.
93. or/91-92
94. 90 not 93
95. limit 94 to conference abstract status
96. 94 not 95
97. limit 96 to english language
98. exp case report/
99. (case adj2 report$).tw.

For peer review only

100. or/98-99
101. 97 not 100
102. limit 101 to (conference paper or "conference review" or editorial or letter or note)
103. 101 not 102

For peer review only

Appendix 4. GLOBAL HEALTH Search Strategy

1. exp eye diseases/
2. exp vision disorders/
3. cataract$.tw.
4. ((intraocular or intra ocular) adj3 lens$).tw.
5. (IOL or IOLs).tw.
6. (visual adj2 (acuit$ or field$)).tw.
7. contrast sensitivity.tw.
8. (depth perception or stereopsis).tw.
9. ((impair$ or decreas$ or declin$) adj3 (vision or visual$ or sight$)).tw.
10. (improv$ adj3 (vision or visual$ or sight$)).tw.
11. ((visual or vision) adj2 function$).tw.
12. ((eye$ or sight or vision or visual$) adj2 (test$ or screen$ or exam$ or diagnos$ or assess$)).tw.
13. or/1-12
14. drivers/
15. vehicles/
16. motor cars/
17. traffic/
18. traffic accidents/
19. (driver$ or driving).tw.
20. (automobile$ or car or cars or vehicle$).tw.
21. (motoring or motorcar or "motor car" or "motor cars").tw.
22. crash$.tw.
23. ((road or traffic) adj2 injur$).tw.
24. ((road or traffic or motor) adj2 (accident$ or incident$)).tw.
25. ((road or traffic or motor) adj2 collision$).tw.
26. or/14-25
27. cohort studies/
28. case-control studies/
29. longitudinal studies/
30. retrospective studies/
31. epidemiology/
32. exp clinical trials/
33. randomized controlled trials/
34. feasibility studies/
35. pilot projects/
36. meta-analysis/
37. systematic reviews/
38. reviews/
39. questionnaires/
40. surveys/
41. epidemiological surveys/
42. risk factors/
43. (population or cohort or observation$ or intervention$ or prospective or retrospective or comparative).tw.
44. (questionnaire$ or survey$).tw.
45. (randomized or randomised or randomly or RCT).tw.
46. (systematic review or meta-analysis).tw.
47. (before adj2 after).tw.
48. (case$ adj2 control$).tw.

For peer review only

49. (cross adj1 section$).tw.
50. or/27-49
51. 13 and 26
52. 50 and 51
53. (animal$ or mouse or mice$ or dog or canine or rat or rats or primate$).ti.
54. (dry eye or cell$ or mutation$ or genes or genome or sequencing).ti.
55. 53 or 54
56. 52 not 55
57. limit 56 to english language
58. case reports/
59. (case adj2 report$).tw.
60. 58 or 59
61. 57 not 60
62. limit 61 to (conference or conference paper or conference proceedings or correspondence or editorial or thesis)
63. 61 not 62

For peer review only

BMJ Open

Associations between vision impairment and driving and the effectiveness of vision-related interventions: protocol for a systematic review and meta-analysis

Journal:	*BMJ Open*
Manuscript ID	bmjopen-2020-040881.R1
Article Type:	Protocol
Date Submitted by the Author:	22-Aug-2020
Complete List of Authors:	Nguyen, Helen; University of New South Wales<br>Di Tanna, Gian Luca; George Institute for Global Health,<br>Coxon, Kristy; Western Sydney University, School of Health Sciences<br>Brown, Julie; George Institute for Global Health, Injury<br>Ren, Kerrie; University of New South Wales<br>Ramke, Jacqueline; LSHTM,<br>Burton, Matthew J; London School of Hygiene and Tropical Medicine<br>Gordon, Iris<br>Zhang, Justine; London School of Hygiene and Tropical Medicine Faculty of Infectious and Tropical Diseases, International Centre for Eye Health, Clinical Research Department<br>Furtado, João; Universidade de São Paulo Faculdade de Medicina de Ribeirão Preto, Division of Ophthalmology<br>Mdala, Shaffi; Queen Elizabeth Central Hospital, Opthalmology Department<br>Kitema, Gatera Fiston; University of Rwanda College of Medicine and Health Sciences, Opthalmology Department<br>Keay, Lisa; University of New South Wales, School of Optometry and Vision Science; The George Institute for Global Health
**Primary Subject Heading**:	Ophthalmology
Secondary Subject Heading:	Public health
Keywords:	PUBLIC HEALTH, OPHTHALMOLOGY, EPIDEMIOLOGY

SCHOLARONE™
Manuscripts

I, the Submitting Author has the right to grant and does grant on behalf of all authors of the Work (as defined in the below author licence), an exclusive licence and/or a non-exclusive licence for contributions from authors who are: i) UK Crown employees; ii) where BMJ has agreed a CC-BY licence shall apply, and/or iii) in accordance with the terms applicable for US Federal Government officers or employees acting as part of their official duties; on a worldwide, perpetual, irrevocable, royalty-free basis to BMJ Publishing Group Ltd ("BMJ") its licensees and where the relevant Journal is co-owned by BMJ to the co-owners of the Journal, to publish the Work in this journal and any other BMJ products and to exploit all rights, as set out in our licence.

The Submitting Author accepts and understands that any supply made under these terms is made by BMJ to the Submitting Author unless you are acting as an employee on behalf of your employer or a postgraduate student of an affiliated institution which is paying any applicable article publishing charge ("APC") for Open Access articles. Where the Submitting Author wishes to make the Work available on an Open Access basis (and intends to pay the relevant APC), the terms of reuse of such Open Access shall be governed by a Creative Commons licence – details of these licences and which Creative Commons licence will apply to this Work are set out in our licence referred to above.

Other than as permitted in any relevant BMJ Author's Self Archiving Policies, I confirm this Work has not been accepted for publication elsewhere, is not being considered for publication elsewhere and does not duplicate material already published. I confirm all authors consent to publication of this Work and authorise the granting of this licence.

For peer review only

Associations between vision impairment and driving and the effectiveness of vision-related interventions: protocol for a systematic review and meta-analysis

Authors: Helen Nguyen[1], Gian Luca Di Tanna[2], Kristy Coxon[3], Julie Brown[2], Kerrie Ren[1], Jacqueline Ramke[4,5], Matthew J Burton[4,6], Iris Gordon[4], Justine H Zhang[4,7], João M. Furtado[8], Shaffi Mdala[9], Gatera Fiston Kitema[10], Lisa Keay[1,2]

Affiliations:

1 School of Optometry and Vision Science, Faculty of Science, The University of New South Wales, NSW, Australia

2 The George Institute for Global Health, Faculty of Medicine, University of New South Wales, NSW, Australia

3 School of Health Sciences, and the Translational Health Research Institute, Western Sydney University, NSW, Australia

4 International Centre for Eye Health, London School of Hygiene & Tropical Medicine, London, United Kingdom, WC1 E7HT

5 School of Optometry and Vision Science, University of Auckland, Auckland, New Zealand

6 Moorfields Eye Hospital, London, UK

7 Manchester Royal Eye Hospital, Manchester, United Kingdom

8 Division of Ophthalmology, Ribeirão Preto Medical School, University of São Paulo, Ribeirão Preto, São Paulo, Brazil

9 Ophthalmology Department, Queen Elizabeth Central Hospital, Blantyre, Malawi

10 Ophthalmology Department, School of Health Sciences, University of Rwanda, Kigali, Rwanda

Corresponding Author: Lisa Keay

Postal Address: School of Optometry and Vision Science, The University of New South Wales, Sydney UNSW NSW 2052, Australia

Email: l.keay@unsw.edu.au

Keywords: visual impairment, road traffic injuries, motor vehicle crashes, driving cessation, driving errors, driving performance, fatal crash involvement

For peer review only - http://bmjopen.bmj.com/site/about/guidelines.xhtml

ABSTRACT

Introduction: Driving is one of the main modes of transport with safe driving requiring a combination of visual, cognitive, and physical skills. With population ageing, the number of people living with vision impairment is set to increase in the decades ahead. Vision impairment may negatively impact an individual's ability to safely drive. The association between vision impairment and motor vehicle crash involvement or driving participation has yet to be systematically investigated. Further, the evidence for the effectiveness of vision-related interventions aimed at decreasing crashes and driving errors has not been synthesised.

Methods and Analysis: A search will be conducted for relevant studies on Medline (Ovid), EMBASE, and Global Health from their inception without date or geographical restrictions. Two investigators will independently screen abstracts and full-texts using Covidence software with conflicts resolved by a third investigator. Data extraction will be conducted on all included studies, and their quality assessed to determine risk of bias using the Joanna Briggs Institute (JBI) Critical Appraisal Tools. Outcome measures include crash risk, driving cessation, and surrogate measures of driving safety (e.g. driving errors and performance). The results of this review will be reported using the Preferred Reporting Items for Systematic Review and Meta-Analysis (PRISMA) guideline. Meta-analysis will be undertaken for outcomes with sufficient data and reported following the Meta-analyses of Observational Studies in Epidemiology (MOOSE) guideline. Where statistical pooling is not feasible or appropriate, narrative summaries will be presented following the Synthesis Without Meta-analysis (SWiM) in systematic reviews guideline.

Ethics and dissemination: This review will only report on published data thus no ethics approval is required. Results will be included in the *Lancet Global Health* Commission on Global Eye Health, published in a peer-reviewed journal, and presented at relevant conferences.

PROSPERO registration number: CRD42020172153

For peer review only

For peer review only - http://bmjopen.bmj.com/site/about/guidelines.xhtml

STRENGTHS AND LIMITATIONS OF THIS STUDY

- Results from this systematic review will present up-to-date evidence for the influence of vision impairment on road traffic injuries (RTIs) and the effectiveness of vision-related interventions.
- As there are no geographic restrictions in the criteria for included studies, this review will capture a large portion of English-language publications on this area with findings applicable to a global context.
- This review will not restrict the age of the target population allowing evidence on the impact of vision impairment and driving to be documented for all age groups.
- This review only looks at published studies in English, so research from non-English speaking countries will be missed, which could introduce bias
- Another potential limitation is that interventions and outcome measures may be highly heterogeneous which will affect the conclusions drawn from the results and prevent meta-analyses to be conducted for select outcomes.

For peer review only

INTRODUCTION

According to the World Health Organisation (WHO),(1) approximately 1.35 million people die each year from road traffic injuries (RTIs), making it the eighth leading cause of death globally. Low- and middle income countries (LMICs) have lower rates of vehicle ownership compared to high-income countries (HIC), but over 90% of RTI fatalities occur in LMICs with the highest death rates in Africa. Individuals from lower socioeconomic backgrounds living in HICs are also more likely to be involved in a road crash resulting in injuries. RTIs make up a major proportion of a country's economic and social burden,(2-4) and account for almost 30% of global injury-related disability.(2) In the face of increasing motorisation, achieving absolute reductions in RTIs is a challenge, especially for vulnerable road users such as pedestrians and users of powered two and three wheeler vehicles. This challenge has a direct impact on the UN's Sustainable Development Goals (SDGs), in particularly Target 3.6 which called for a halving of global road deaths by 2020, and Target 11.2 which called for safe and sustainable transport systems, especially for vulnerable road users.(5)

Motor vehicle crashes (MVCs), and by extension RTIs, however, are preventable. Using the Haddon Matrix, an early theory describing the multifactorial nature of RTIs, MVCs are understood to involve host (human), agent (vehicles and equipment), and environmental (physical and socioeconomic) factors(6). This theory has since been used to build the Safe System Approach endorsed in the United Nations Road Safety Collaboration's Decade of Action for Road Safety (2011-2020).(7) In brief, the Safe System Approach aims to prevent MVCs which result in serious injuries or death by addressing four main pillars of focus: 1) safe roads, 2) safe speeds, 3) safe people and 4) safe vehicles.(8) Road safety programs, such as the *Bloomberg Initiative for Global Road Safety (2015-2019)* focus on improving road safety through legislation in LMICs,(9) thus addressing the environmental and agent risks of RTIs. Beyond road infrastructure and vehicle quality, human driving behaviours or 'human factors' also contribute to RTI rates and are an intrinsic part of the Safe System Approach. Safe driving requires individuals to have a range of physical, visual, and cognitive skills. In addition to specific eye diseases, age-related functional declines across a range of domains, including vision, can reduce confidence in driving ability.(10) Poor visual acuity and contrast sensitivity, visual field loss, and glare sensitivity have all been identified as potential factors contributing to poor driving performance and increased MVCs.(11)

Due to the high visual demands needed to drive safely, many countries have set federal and or state-specific standards for vision, mostly for visual acuity. Most countries accept that a visual acuity of at least 6/12 (0.50, 20/40) in the better eye as the requirement for driving. This threshold dictates jurisdictional control used to identify individuals with vision impairment and restrict their access to

For peer review only - http://bmjopen.bmj.com/site/about/guidelines.xhtml

driving privileges. However, a systematic review by Dobbs (2008) suggested that licencing policies aimed at identifying at-risk older drivers may not be effective in decreasing crash rates.(12) This may be because policies which govern licensure and vision screening vary significantly between and within countries. Further, evidence on their effectiveness is inconclusive.(13) Conversely, in-person renewal policies which include vision tests completed at licence renewal centres, have been shown to reduce crash rates in older drivers.(14) An American study analysing data from the National Highway Traffic Safety Administration Fatal Accident Reporting System found drivers aged 70 years and older who underwent visual acuity examinations during their licence renewals had lower fatal crash risks than their non-vision tested peers (RR 0.93; 95% Cl 0.89 – 0.97).(15) However, the literature remains divided in its support for using visual acuity alone as a predictor of MVC involvement and high-risk driving behaviours.(16) A Cochrane review, updated twice, examined the benefits of different vision screening procedures, such as visual acuity, visual field (central or peripheral), contrast sensitivity, and useful field of view (UFOV) tests, in randomised-controlled trials (RCTs) aimed at preventing RTIs and fatalities in older drivers.(13, 17) Unfortunately, no RCTs met the inclusion criteria for the review at the time these reviews were conducted.

There is substantial literature investigating how vision impairment, and other aspects of function, affect road safety. Measures of driving safety have included indirect measures such as performance on driving simulators, on-road driving assessments, naturalistic driving or in-vehicle monitoring as well as direct measures of RTI and MVC rates from self-report or administrative datasets.(18, 19) However, the evidence for the influence of vision loss on MVCs and the corresponding benefits of interventions to restore vision have not been systematically evaluated. Since older drivers have higher crash involvement(20) and greater prevalence of eye diseases due to natural age-related declines in vision,(21) most research investigate older drivers and their risks of crashes and injuries. However, it is important to document the impact of vision impairment across all age groups. Further, information is also needed about specific eye diseases and types of vision impairment to inform interventions to screen for poor vision in drivers, and interventions to rehabilitate vision, thereby enhancing driver safety and continued ability to drive. This is especially important for older adults who rely on driving to remain independent and connected with their community. The loss of the ability to drive and the eventual retirement from driving has been linked to higher symptoms of depression and poorer health in older adults.(22)

The aim of this systematic review is to: 1) describe the associations between vision impairment and risk of road crash involvement across the lifespan, and 2) evaluate vision-related interventions to reduce crash risk. Secondary outcomes are driving cessation and surrogate measures of crash risk such as on-road driving errors.

For peer review only - http://bmjopen.bmj.com/site/about/guidelines.xhtml

METHODS AND ANALYSIS

This systematic review protocol was drafted using the International Prospective Register of Systematic Reviews (PROSPERO) as a guideline and registered in PROSPERO (28/04/2020; CRD42020172153 [https://www.crd.york.ac.uk/prospero/display_record.php?ID=CRD42020172153]). Any changes to the protocol will be updated in PROSPERO. The protocol is prepared in accordance to the Preferred Reporting Items for Systematic review and Meta-Analysis Protocols (PRISMA-P) statement (Appendix no. 1).(23)

Eligibility Criteria

This review will include human studies in the English language with full-text available. Unlike the two previous Cochrane reviews which only included RCTs, this review will consider both interventional (RCTs and quasi-experimental) and observational (cohort, cross-sectional, and case-control) studies. Systematic reviews will be included if meta-analysis was performed. For systematic reviews without meta-analysis, the reference list will be examined for potentially relevant articles, but the systematic review itself will not be included. All literature reviews, commentary articles, dissertations, abstracts, editorials, and conference presentations will be excluded.

All studies must report on at least one of the outcome variables, described in the following section, which include MVC involvement and surrogate measures of driving safety such as driving errors and performance scores and driving cessation. Studies investigating either self-regulatory behaviours, such as night driving avoidance and decreasing travel mileage, or self-reported measures of driving safety, will be excluded. To obtain data on driving scores and performance, studies using on-road driving tests, which include closed-circuit routes and those combining both closed and real-road driving tracks, and naturalistic driving with in-vehicle monitoring will be included. Even though closed-circuits may not reflect true on-road driving conditions, tests for common driving maneuverers such as road signage recognition, hazard recognition and avoidance, reversing, and gap perception are able to be recreated on these routes.(24) Driving errors and driving performance scores on the on-road driving tests can come from fitted in-vehicle monitoring technologies or trained observers. Studies which used driving simulators will be excluded as these are laboratory studies with only indirect measures of driving performance. MVC involvement cannot be measured and real-life driving experiences, such as limited exposure to driving at night, in bad weather, or during rush hour, may not be reflected in a simulation.(25) Additionally, the validity of driving simulator results are highly dependent upon the type of simulation program used and what kind of driving manoeuvre is being investigated.(26) As this review is interested in the MVC involvement and

For peer review only

driving abilities of individuals who drive and their habitual vision, studies which simulate impairments in vision will also be excluded.

The population of focus will be drivers of four-wheeled motorised vehicles such as cars, buses, and trucks. Unlike the two Cochrane reviews mentioned above which only focused on older drivers, this review will include drivers of all ages. Studies of drivers who have specific medical conditions (e.g. dementia, epilepsy, stroke, and history of medical events such as syncope), or vision difficulties due to other medical factors (e.g. hemianopia caused by brain damage) will not be included. Similarly, articles where vision status is not reported will be excluded.

Exposures in the included studies will encompass all types of vision impairment including visual acuity, contrast sensitivity, visual field loss as well as impairments associated with specific eye diseases including but not limited to glaucoma, cataracts, aged-related macular degeneration (AMD), diabetic retinopathy (DR), stereopsis disorders, and colour vision deficiencies. Vision impairments can be categorised by the specific eye diseases or by specific measures of vision which can negatively impact normal everyday functioning. Even though it is not necessary for all included studies to report on vision-related interventions, studies which do report on interventions can include procedures such as vision screening, refractive correction, cataract surgery or other measures to restore and improve vision of drivers in order to maintain driving participation, promote safe driving and reduce risk of crash involvement. The exposure comparators of included studies will be drivers who either do not have a vision impairment or have not received a vision-related intervention, within a timeframe chosen by the study in question.

Outcome Measures

The primary outcome measure is MVC involvement including fatal MVC involvement. Data on crash involvement and its severity can either come from self-reported surveys or data linkage with government and/or hospital records. Data from self-reported surveys will ensure that MVCs which were not serious enough to warrant a police or hospital report will be also be included.

Driving cessation and surrogate measures of driving safety will be the secondary outcomes. The surrogate measures of driving safety can include scores of driving performance from on-road driving tests or 'naturalistic' in-vehicle monitoring looking at manoeuvres such as lane keeping, braking, and abidance of road signage like traffic lights, stop and give way signs. To account for differences in the criteria used by trained observers to evaluate the driving performance scores on on-road driving tests, a pass/fail threshold for driving performance scores specific to this review will be decided upon by all investigators in order to synthesise results.

For peer review only - http://bmjopen.bmj.com/site/about/guidelines.xhtml

Search Strategy

Electronic database search will be conducted by the Cochrane Eyes and Vision Information Specialist (IG) on Medline (Ovid), EMBASE, and Global Health from their inception to March 2020. Appendices 2, 3, and 4 shows the search strategies for Medline, EMBASE, and Global Health, respectively. Additional potentially relevant studies will be sought by experts in the field by checking the reference lists and citations of included studies, and checking the reference list of narrative systematic reviews identified in the search.

Data Collection and Analysis

Data Management and Selection

Each title and abstract will be screened by two investigators independently (from HN, KR, JR, JZ, SM, JF, GFK) using Covidence systematic review management software (Veritas Health Innovation, Melbourne, Australia; available at https://www.covidence.org/home). Full-text review of potentially relevant articles will then be conducted by two investigators independently. Discrepancies will be discussed and resolved via consultation with a third investigator.

Data Extraction

Data extraction will be completed independently by two investigators (from among the same seven investigators). Data from included studies will be extracted using adaptions of the Joanna Briggs Institute (JBI) template for systematic reviews and observational studies (including cohort, cross-sectional, and case-control studies).(27) Adapted Cochrane templates will be used to extract data from randomized controlled trials and quasi-experimental studies.(28)

Quality Assessment

A quality assessment to determine an overall risk of bias will be carried out on all included studies independently by two investigators (from the seven investigators mentioned previously). Conflicts will be resolved by a third investigator. Relevant JBI critical appraisal tools will be used to evaluate randomised controlled trials, quasi-experimental studies, systematic reviews, cohort studies, cross-sectional studies, and case-control studies.(29)

Data Synthesis Strategy

Measures of association between vision impairment/vision related interventions and MVC involvement, driving cessation and surrogate measures of driving safety will be summarized according to the outcome measures reported in the primary studies. In particular, appropriate hazard ratio (HR), risk ratio (RR), and odds ratio (OR) for binary data and (standardized) mean

For peer review only - http://bmjopen.bmj.com/site/about/guidelines.xhtml

differences for continuous data will be statistically pooled. When the same outcome is reported as dichotomous data in some studies and as continuous data in others, these studies will be pooled by expressing the ORs as standardized mean differences and vice versa.(30) P-values of the driving outcomes will also be reported where appropriate.

Where it is not possible or suitable to statistically pool the studies, a narrative summary of the findings will be used instead. Narrative summaries will follow the Synthesis Without Meta-analysis (SWiM) reporting guidelines.(31) Heterogeneity across all included studies with sufficient data will be assessed clinically, methodologically and statistically. Clinical heterogeneity will be accessed by comparing the differences between the participant characteristics (e.g. age, sex, eye disease), interventions and outcomes measured. The design and quality of included studies will be compared to access methodological heterogeneity. Statistical heterogeneity across studies will be explored by formal statistical test of heterogeneity, subgroup analyses and, if feasible, by meta-regression. Inconsistency of the effect sizes across the studies will be assessed by the proportion of variability in the effect sizes of the included studies due to heterogeneity (and not by sampling error) using I^2. Estimates will be pooled using random effects models with fixed effect models results also reported regardless of the values of I^2, and prediction intervals to allow for expected effects of future studies to be extrapolated based upon the current evidence.(32)

The following outcomes will be assessed using meta-analysis where feasible according to data availability: crash involvement, driving cessation, and surrogate measures of un-safe driving i.e. driving errors and driving performance. Furthermore meta-analyses for each of the different eye diseases, and studies from LMIC settings will also be performed independently for each outcome of interest if possible. As there is no age restriction on the focus population, results on age will be synthesised by assessing specific subgroup analysis and/or meta-regression which may partially explain heterogeneity across studies in the pooled effect size. The Meta-analysis of Observational Studies in Epidemiology (MOOSE) guidelines will be used to guide reporting.(33) The Grading of Recommendations Assessment, Development and Evaluation (GRADE) approach will be used to assess the quality of evidence in the meta-analyses.(34)

Sensitivity analysis will be performed on low risk of bias studies whilst the meta-analysis will include all studies. This will assist with verifying the strength of the study findings and to assess how different methodologies, sample sizes and statistical analyses have affected this study's results. Furthermore, funnel plots will be used to assess publication bias.

Corresponding authors from publications dated 2010 onwards with missing data of potential use will be approached via email, up to a maximum of 3 attempts, to request further information. Any

For peer review only - http://bmjopen.bmj.com/site/about/guidelines.xhtml

unobtainable data will be noted alongside all attempts to contact the respective authors. Even though only available data will be used for the meta-analysis, the effects of any missing data will be considered and their effects discussed in the overall final review.

Patient and Public Involvement

This review will only be looking at existing published literature. No patient or public involvement is currently planned for the design and execution of this review, however public participation may be sought for this review's dissemination.

ETHICS AND DISSEMINATION

As this review will only be focusing on currently published literature, ethics approval is not required. Results from this systematic review will be published in an open peer-reviewed journal and will form part of the ongoing *Lancet Global Health* Commission on Global Eye Health.(35) Where relevant, it will also be presented at conferences.

DISCUSSION

Significance of this review

The findings of this systematic review may influence future road safety policies on driving and care for drivers with vision impairment. By understanding the visual factors contributing to MVCs, , vision-related screening tests for licencing may be reconsidered and updated to increase relevance to driving safety. As mentioned previously, most reviews on driving with vision impairment have been limited to older drivers and the effects of different licencing renewal procedures on their ability to drive. Even though older drivers are at higher risk,(21) this review will seek to capture data on driving and vision impairment for all age groups.

The eligibility criteria for included studies for this review will ensure that global data on vision and driving will be captured. Currently, MVC-related societal burdens and injury- related disability burdens in LMICs are poorly understood which may partially explain why cost-effective interventions in these countries are rarely undertaken.(2) LMICs tend to focus on legislative interventions, followed by education/training workshops, public awareness campaigns, enforcement measures, speed control and infrastructure improvements.(36) Current data on human factors specifically related to vision impairment in LMICs reported in this review may inform future evidence-based policies on licencing and/or screening policies to address these gaps.

Results from this review may also provide additional evidence on the impact of eye-disease specific interventions on quality of life factors, especially those related to driving and the ability to drive. Interventions to improve and optimise vision are needed for drivers, in recognition of the important

For peer review only - http://bmjopen.bmj.com/site/about/guidelines.xhtml

of continued safe driving. This greater awareness in turn will also provide evidence for policies around road safety for individuals with vision impairments.

For peer review only

For peer review only - http://bmjopen.bmj.com/site/about/guidelines.xhtml

REFERENCES

1 World Health Organisation. Road Traffic Injuries 2020; https://www.who.int/news-room/fact-sheets/detail/road-traffic-injuries (accessed April 2020)

2 Bachani AM, Peden M, Gururaj G, et al. Road Traffic Injuries. In: Mock CN, Nugent R, Kobusingye O, Smith KR, eds. Injury Prevention and Environmental Health. Washington (DC): The International Bank for Reconstruction and Development / The World Bank © 2017 International Bank for Reconstruction and Development / The World Bank.; 2017.

3 Chen S, Kuhn M, Prettner K, et al. The global macroeconomic burden of road injuries: estimates and projections for 166 countries. *Lancet Planet Health* 2019;3(9):e390-e8. doi: 10.1016/S2542-5196(19)30170-6

4 Wijnen W, Stipdonk H. Social costs of road crashes: An international analysis. *Accid Anal Prev* 2016;94:97-106. doi: 10.1016/j.aap.2016.05.005 [published Online First: 3 June 2016]

5 United Nations. #Envision2030: 17 goals to transform the world for persons with disabilities; https://www.un.org/development/desa/disabilities/envision2030.html (accessed April 2020)

6 Haddon W, Jr. Energy damage and the ten countermeasure strategies. *J Trauma* 1973;13(4):321-31. doi: 10.1177/001872087301500407

7 The Department of Infrastructure, Regional Development and Communications: Australian Government. Decade of Action for Road Safety 2011-2020 2014; https://www.infrastructure.gov.au/roads/safety/decade_of_action/index.aspx (accessed April 2020)

8 Towards Zero Foundation. The Safe System; http://www.towardszerofoundation.org/thesafesystem/ (accessed 30 July 2020)

9 Global Road Safety Partnership. Bloomberg Initiative for Global Road Safety; https://www.grsproadsafety.org/programmes/bloomberg-initiative-global-road-safety/ (accessed April 2020)

10 Miller SM, Taylor-Piliae RE, Insel KC. The association of physical activity, cognitive processes and automobile driving ability in older adults: A review of the literature. *Geriatr Nurs* 2016;37(4):313-20. doi: 10.1016/j.gerinurse.2016.05.004 [published Online First: 31 May 2016]

11 Rubin GS, Ng ES, Bandeen-Roche K, et al. A prospective, population-based study of the role of visual impairment in motor vehicle crashes among older drivers: the SEE study. *Invest Ophthalmol Vis Sci* 2007;48(4):1483-91. doi: 10.1167/iovs.06-0474

12 Dobbs BM. Aging baby boomers--a blessing or challenge for driver licensing authorities. *Traffic Inj Prev* 2008;9(4):379-86. doi: 10.1080/15389580802045823

13 Desapriya E, Harjee R, Brubacher J, et al. Vision screening of older drivers for preventing road traffic injuries and fatalities. *Cochrane Database Syst Rev* 2014(2):Cd006252. doi: 10.1002/14651858.CD006252.pub3

For peer review only - http://bmjopen.bmj.com/site/about/guidelines.xhtml

14 Grabowski DC, Campbell CM, Morrisey MA. Elderly licensure laws and motor vehicle fatalities. *JAMA* 2004;291(23):2840-6. doi: 10.1001/jama.291.23.2840

15 Levy DT, Vernick JS, Howard KA. Relationship between driver's license renewal policies and fatal crashes involving drivers 70 years or older. *JAMA* 1995;274(13):1026-30. doi: 0.1001/jama.1995.03530130032024

16 Owsley C, McGwin G, Jr. Vision impairment and driving. *Surv Ophthalmol* 1999;43(6):535-50.

17 Subzwari S, Desapriya E, Babul-Wellar S, et al. Vision screening of older drivers for preventing road traffic injuries and fatalities. *Cochrane Database Syst Rev* 2009(1):Cd006252. doi: 10.1002/14651858.CD006252.pub2

18 Imprialou M, Quddus M. Crash data quality for road safety research: Current state and future directions. *Accid Anal Prev* 2019;130:84-90. doi: 10.1016/j.aap.2017.02.022

19 Kaye SA, Lewis I, Freeman J. Comparison of self-report and objective measures of driving behavior and road safety: A systematic review. *J Safety Res* 2018;65:141-51. doi: 10.1016/j.jsr.2018.02.012

20 Owsley C, Ball K, Sloane ME, et al. Visual/cognitive correlates of vehicle accidents in older drivers. *Psychol Aging* 1991;6(3):403-15. doi: 10.1037//0882-7974.6.3.403

21 Wood JM, Black AA. Ocular disease and driving. *Clin Exp Optom*. 2016;99(5):395-401. doi: 10.1111/cxo.12391 [published Online First: 7 May 2016]

22 Ragland DR, Satariano WA, MacLeod KE. Driving cessation and increased depressive symptoms. *J Gerontol A Biol Sci Med Sci* 2005;60(3):399-403. doi: 10.1093/gerona/60.3.399

23 Shamseer L, Moher D, Clarke M, et al. Preferred reporting items for systematic review and meta-analysis protocols (PRISMA-P) 2015: elaboration and explanation. *BMJ* 2015;350:g7647. doi: 10.1136/bmj.g7647

24 Wood JM. Age and visual impairment decrease driving performance as measured on a closed-road circuit. *Hum factors*. 2002;44(3):482-94. doi: 10.1518/0018720024497664

25 Keay L, West SK. Vision and Driving Performance in Elderly. In: Scholl HPN, Massof RW, West SK eds. Ophthalmology and the Ageing Society. Berlin Heidelberg: Springer-Verlag 2013:193-207.

26 Shechtman O, Classen S, Awadzi K, et al. Comparison of driving errors between on-the-road and simulated driving assessment: a validation study. *Traffic Inj Prev* 2009;10(4):379-85. doi: 10.1080/15389580902894989

27 The Joanna Briggs Institute. Joanna Briggs Institute Reviewer's Manual. 2017. Available from: https://reviewersmanual.joannabriggs.org/ (accessed April 2020)

28 Cochrane: Developmental PaLP. Data Extraction Forms 2014 [Available from: https://dplp.cochrane.org/data-extraction-forms (accessed April 2020).

29 University of Adelaide. Critical Appraisal Tools; https://joannabriggs.org/ebp/critical_appraisal_tools (assessed April 2020)

For peer review only - http://bmjopen.bmj.com/site/about/guidelines.xhtml

30 Anzures-Cabrera J, Sarpatwari A, Higgins JP. Expressing findings from meta-analyses of continuous outcomes in terms of risks. *Stat Med* 2011;30(25):2967-85. doi: 10.1002/sim.4298 [published Online First: 8 August 2011]

31 Campbell M, McKenzie JE, Sowden A, et al. Synthesis without meta-analysis (SWiM) in systematic reviews: reporting guideline. *BMJ* 2020;368:l6890. doi: 10.1136/bmj.l6890

32 IntHout J, Ioannidis JP, Rovers MM, et al. Plea for routinely presenting prediction intervals in meta-analysis. *BMJ Open* 2016;6(7):e010247. doi: 10.1136/bmjopen-2015-010247

33 Stroup DF, Berlin JA, Morton SC, et al. Meta-analysis of observational studies in epidemiology: a proposal for reporting. Meta-analysis Of Observational Studies in Epidemiology (MOOSE) group. *JAMA* 2000;283(15):2008-12. doi: 10.1001/jama.283.15.2008

34 Iorio A, Spencer FA, Falavigna M, et al. Use of GRADE for assessment of evidence about prognosis: rating confidence in estimates of event rates in broad categories of patients. *BMJ* 2015;350:h870. doi: 10.1136/bmj.h870

35 Burton MJ, Faal HB, Ramke J, et al. Announcing The Lancet Global Health Commission on Global Eye Health. *Lancet Glob Health* 2019;7(12):e1612-e3. doi: 10.1016/S2214-109X(19)30450-4 [published Online First: 9 October 2019]

36 Staton C, Vissoci J, Gong E, et al. Road Traffic Injury Prevention Initiatives: A Systematic Review and Metasummary of Effectiveness in Low and Middle Income Countries. *PloS One* 2016;11(1):e0144971. doi: 10.1371/journal.pone.0144971

For peer review only

For peer review only - http://bmjopen.bmj.com/site/about/guidelines.xhtml

Authors' Contributions: HN, LK, and JR conceived the idea for the review. HN drafted and revised the protocol with suggestions from LK, JR, JB, KC, GLdT, KR, MJB, JZ, IG, JFM, SM, and GFK who reviewed the protocol and provided feedback on the draft. IG constructed the search. The final version of the manuscript was approved by all authors.

Funding Statement: MJB is supported by the Wellcome Trust (207472/Z/17/Z). JR is a Commonwealth Rutherford Fellow, funded by the UK government through the Commonwealth Scholarship Commission in the UK. The Lancet Global Health Commission on Global Eye Health is supported by The Queen Elizabeth Diamond Jubilee Trust, The Wellcome Trust, Sightsavers, The Fred Hollows Foundation, The SEVA Foundation, The British Council for the Prevention of Blindness and Christian Blind Mission.

Acknowledgements: None.

Competing Interests: None declared.

Number of Tables: 0

Number of Figures: 0

Data Sharing Statement: Data generated from this review will be available upon reasonable request from HN.

Word Count: 3349

For peer review only

Appendix 1. Preferred Reporting Items for Systematic review and Meta-analysis Protocols (PRISMA-P) 2015 Checklist

Section and topic	Item No	Checklist item	Page Number
**ADMINISTRATIVE INFORMATION**			
Title:			
Identification	1a	Identify the report as a protocol of a systematic review	1
Update	1b	If the protocol is for an update of a previous systematic review, identify as such	N/A
Registration	2	If registered, provide the name of the registry (such as PROSPERO) and registration number	2, 6
Authors:			
Contact	3a	Provide name, institutional affiliation, e-mail address of all protocol authors; provide physical mailing address of corresponding author	1
Contributions	3b	Describe contributions of protocol authors and identify the guarantor of the review	8, 15
Amendments	4	If the protocol represents an amendment of a previously completed or published protocol, identify as such and list changes; otherwise, state plan for documenting important protocol amendments	N/A
Support:			
Sources	5a	Indicate sources of financial or other support for the review	15
Sponsor	5b	Provide name for the review funder and/or sponsor	15
Role of sponsor or funder	5c	Describe roles of funder(s), sponsor(s), and/or institution(s), if any, in developing the protocol	15
**INTRODUCTION**			
Rationale	6	Describe the rationale for the review in the context of what is already known	4, 5
Objectives	7	Provide an explicit statement of the question(s) the review will address with reference to participants, interventions, comparators, and outcomes (PICO)	5
**METHODS**			
Eligibility criteria	8	Specify the study characteristics (such as PICO, study design, setting, time frame) and report characteristics (such as years considered, language, publication status) to be used as criteria for eligibility for the review	6, 7
Information sources	9	Describe all intended information sources (such as electronic databases, contact with study authors, trial registers or other grey literature sources) with planned dates of coverage	6, 8

For peer review only

Search strategy	10	Present draft of search strategy to be used for at least one electronic database, including planned limits, such that it could be repeated	Appendix no. 2-4
Study records:			
Data management	11a	Describe the mechanism(s) that will be used to manage records and data throughout the review	8
Selection process	11b	State the process that will be used for selecting studies (such as two independent reviewers) through each phase of the review (that is, screening, eligibility and inclusion in meta-analysis)	8
Data collection process	11c	Describe planned method of extracting data from reports (such as piloting forms, done independently, in duplicate), any processes for obtaining and confirming data from investigators	8
Data items	12	List and define all variables for which data will be sought (such as PICO items, funding sources), any pre-planned data assumptions and simplifications	6, 7, 8
Outcomes and prioritization	13	List and define all outcomes for which data will be sought, including prioritization of main and additional outcomes, with rationale	7
Risk of bias in individual studies	14	Describe anticipated methods for assessing risk of bias of individual studies, including whether this will be done at the outcome or study level, or both; state how this information will be used in data synthesis	8, 9
Data synthesis	15a	Describe criteria under which study data will be quantitatively synthesised	8, 9
	15b	If data are appropriate for quantitative synthesis, describe planned summary measures, methods of handling data and methods of combining data from studies, including any planned exploration of consistency (such as I^2, Kendall's τ)	8, 9
	15c	Describe any proposed additional analyses (such as sensitivity or subgroup analyses, meta-regression)	8, 9
	15d	If quantitative synthesis is not appropriate, describe the type of summary planned	2, 9
Meta-bias(es)	16	Specify any planned assessment of meta-bias(es) (such as publication bias across studies, selective reporting within studies)	8, 9
Confidence in cumulative evidence	17	Describe how the strength of the body of evidence will be assessed (such as GRADE)	9

For peer review only

Appendix 2. MEDLINE (OVID) search strategy

1. exp Eye Diseases/
2. exp Cataract Extraction/
3. Lens Implantation, Intraocular/
4. Lenses, Intraocular/
5. cataract$.tw.
6. ((intraocular or intra ocular) adj3 lens$).tw.
7. (IOL or IOLs).tw.
8. Vision Tests/
9. Visual Acuity/
10. exp Refractive Errors/
11. Visual Fields/
12. Visual Field Tests/
13. Contrast Sensitivity/
14. Depth Perception/
15. (visual adj2 (acuit$ or field$)).tw.
16. contrast sensitivity.tw.
17. (depth perception or stereopsis).tw.
18. ((impair$ or decreas$ or declin$) adj3 (vision or visual$ or sight$)).tw.
19. (improv$ adj3 (vision or visual$ or sight$)).tw.
20. ((visual or vision) adj2 function$).tw.
21. exp Vision, Ocular/
22. Vision Screening/
23. or/1-22
24. Mass Screening/
25. ((eye$ or sight or vision or visual$) adj2 (test$ or screen$ or exam$ or diagnos$ or assess$)).tw
26. 24 and 25
27. 23 or 26
28. exp Motor Vehicles/
29. exp Automobile Driving/
30. Accidents, Traffic/
31. (driver$ or driving).tw.
32. (automobile$ or car or cars or vehicle$).tw.
33. (motoring or motorcar or "motor car" or "motor cars").tw.
34. crash$.tw.
35. ((road or traffic) adj2 injur$).tw.
36. ((road or traffic or motor) adj2 (accident$ or incident$)).tw.
37. ((road or traffic or motor) adj2 collision$).tw.
38. or/28-37
39. epidemiologic studies/ or case-control studies/ or cohort studies/ or observational study/ or follow-up studies/ or longitudinal studies/ or prospective studies/ or retrospective studies/ or controlled before-after studies/ or cross-sectional studies/ or historically controlled study/ or interrupted time series analysis/
40. epidemiologic methods/ or focus groups/ or interviews as topic/ or exp "surveys and questionnaires"/
41. epidemiologic research design/ or control groups/ or cross-over studies/ or double-blind method/ or meta-analysis as topic/ or network meta-analysis/ or random allocation/ or single-blind method/
42. epidemiologic methods/ or clinical trials as topic/ or feasibility studies/ or multicenter studies as topic/ or pilot projects/ or sampling studies/ or twin studies as topic/

For peer review only

43. randomized controlled trial/ or controlled clinical trials as topic/ or randomized controlled trials as topic/
44. comparative study/ or evaluation studies/ or meta-analysis/ or review/ or multicenter study/ or "systematic review"/ or validation studies/
45. health surveys/
46. outcome assessment, health care/
47. risk factors/
48. self report/
49. (population or cohort or observation$ or intervention$ or prospective or retrospective or comparative).tw.
50. (questionnaire$ or survey$).tw.
51. (randomized or randomised or randomly or RCT).tw.
52. (systematic review or meta-analysis).tw.
53. (before adj2 after).tw.
54. (case$ adj2 control$).tw.
55. (cross adj1 section$).tw.
56. or/39-55
57. 27 and 38
58. 56 and 57
59. vehicle-controlled.tw.
60. (vehicle adj3 inject$).tw.
61. 59 or 60
62. 58 not 61
63. (animal$ or mouse or mice$ or dog or canine or rat or rats or primate$).ti.
64. (dry eye or cell$ or mutation$ or genes or genome or sequencing).ti.
65. or/63-64
66. 62 not 65
67. limit 66 to english language
68. exp case reports/
69. (case adj2 report$).tw.
70. 68 or 69
71. 67 not 70
72. limit 71 to (editorial or letter)
73. 71 not 72

For peer review only

Appendix 3. EMBASE Search Strategy

1. exp eye disease/
2. exp cataract extraction/
3. lens implantation/
4. lens implant/
5. cataract$.tw.
6. ((intraocular or intra ocular) adj3 lens$).tw.
7. (IOL or IOLs).tw.
8. vision test/
9. visual acuity/
10. refractive error/
11. visual field/
12. perimetry/
13. contrast sensitivity/
14. depth perception/
15. (visual adj2 (acuit$ or field$)).tw.
16. contrast sensitivity.tw.
17. (depth perception or stereopsis).tw.
18. ((impair$ or decreas$ or declin$) adj3 (vision or visual$ or sight$)).tw.
19. (improv$ adj3 (vision or visual$ or sight$)).tw.
20. ((visual or vision) adj2 function$).tw.
21. vision/
22. or/1-21
23. mass screening/
24. ((eye$ or sight or vision or visual$) adj2 (test$ or screen$ or exam$ or diagnos$ or assess$)).tw.
25. 23 and 24
26. 22 or 25
27. exp car driving/
28. exp motor vehicle/
29. traffic accident/
30. (driver$ or driving).tw.
31. (automobile$ or car or cars or vehicle$).tw.
32. (motoring or motorcar or "motor car" or "motor cars").tw.
33. crash$.tw.
34. ((road or traffic) adj2 injur$).tw.
35. ((road or traffic or motor) adj2 (accident$ or incident$)).tw.
36. ((road or traffic or motor) adj2 collision$).tw.
37. or/27-36
38. study design/
39. controlled clinical trial/
40. case control study/
41. cohort analysis/
42. observational study/
43. follow up/
44. longitudinal study/
45. prospective study/
46. retrospective study/
47. epidemiology/
48. cross-sectional study/
49. control group/

For peer review only

50. crossover procedure/
51. "meta analysis (topic)"/
52. network meta-analysis/
53. randomization/
54. single blind procedure/
55. double blind procedure/
56. "clinical trial (topic)"/
57. "controlled clinical trial (topic)"/
58. "randomized controlled trial (topic)"/
59. "multicenter study (topic)"/
60. feasibility study/
61. pilot study/
62. comparative study/
63. evaluation study/
64. multicenter study/
65. randomized controlled trial/
66. meta analysis/
67. "systematic review"/
68. validation study/
69. interview/
70. questionnaire/
71. outcome assessment/
72. "systematic review (topic)"/
73. health survey/
74. risk factor/
75. self report/
76. evidence based practice/
77. (population or cohort or observation$ or intervention$ or prospective or retrospective or comparative).tw.
78. (questionnaire$ or survey$).tw.
79. (randomized or randomised or randomly or RCT).tw.
80. (systematic review or meta-analysis).tw.
81. (before adj2 after).tw.
82. (case$ adj2 control$).tw.
83. (cross adj1 section$).tw.
84. or/38-83
85. 26 and 37
86. 84 and 85
87. vehicle-controlled.tw.
88. (vehicle adj3 inject$).tw.
89. or/87-88
90. 86 not 89
91. (animal$ or mouse or mice$ or dog or canine or rat or rats or primate$).ti.
92. (dry eye or cell$ or mutation$ or genes or genome or sequencing).ti.
93. or/91-92
94. 90 not 93
95. limit 94 to conference abstract status
96. 94 not 95
97. limit 96 to english language
98. exp case report/
99. (case adj2 report$).tw.

For peer review only

100. or/98-99
101. 97 not 100
102. limit 101 to (conference paper or "conference review" or editorial or letter or note)
103. 101 not 102

For peer review only

Appendix 4. GLOBAL HEALTH Search Strategy

1. exp eye diseases/
2. exp vision disorders/
3. cataract$.tw.
4. ((intraocular or intra ocular) adj3 lens$).tw.
5. (IOL or IOLs).tw.
6. (visual adj2 (acuit$ or field$)).tw.
7. contrast sensitivity.tw.
8. (depth perception or stereopsis).tw.
9. ((impair$ or decreas$ or declin$) adj3 (vision or visual$ or sight$)).tw.
10. (improv$ adj3 (vision or visual$ or sight$)).tw.
11. ((visual or vision) adj2 function$).tw.
12. ((eye$ or sight or vision or visual$) adj2 (test$ or screen$ or exam$ or diagnos$ or assess$)).tw.
13. or/1-12
14. drivers/
15. vehicles/
16. motor cars/
17. traffic/
18. traffic accidents/
19. (driver$ or driving).tw.
20. (automobile$ or car or cars or vehicle$).tw.
21. (motoring or motorcar or "motor car" or "motor cars").tw.
22. crash$.tw.
23. ((road or traffic) adj2 injur$).tw.
24. ((road or traffic or motor) adj2 (accident$ or incident$)).tw.
25. ((road or traffic or motor) adj2 collision$).tw.
26. or/14-25
27. cohort studies/
28. case-control studies/
29. longitudinal studies/
30. retrospective studies/
31. epidemiology/
32. exp clinical trials/
33. randomized controlled trials/
34. feasibility studies/
35. pilot projects/
36. meta-analysis/
37. systematic reviews/
38. reviews/
39. questionnaires/
40. surveys/
41. epidemiological surveys/
42. risk factors/
43. (population or cohort or observation$ or intervention$ or prospective or retrospective or comparative).tw.
44. (questionnaire$ or survey$).tw.
45. (randomized or randomised or randomly or RCT).tw.
46. (systematic review or meta-analysis).tw.
47. (before adj2 after).tw.
48. (case$ adj2 control$).tw.

For peer review only

49. (cross adj1 section$).tw.
50. or/27-49
51. 13 and 26
52. 50 and 51
53. (animal$ or mouse or mice$ or dog or canine or rat or rats or primate$).ti.
54. (dry eye or cell$ or mutation$ or genes or genome or sequencing).ti.
55. 53 or 54
56. 52 not 55
57. limit 56 to english language
58. case reports/
59. (case adj2 report$).tw.
60. 58 or 59
61. 57 not 60
62. limit 61 to (conference or conference paper or conference proceedings or correspondence or editorial or thesis)
63. 61 not 62

For peer review only

BMJ Open

Associations between vision impairment and driving and the effectiveness of vision-related interventions: protocol for a systematic review and meta-analysis

Journal:	*BMJ Open*
Manuscript ID	bmjopen-2020-040881.R2
Article Type:	Protocol
Date Submitted by the Author:	28-Sep-2020

Complete List of Authors:	Nguyen, Helen; University of New South Wales<br>Di Tanna, Gian Luca; George Institute for Global Health,<br>Coxon, Kristy; Western Sydney University, School of Health Sciences<br>Brown, Julie; George Institute for Global Health, Injury<br>Ren, Kerrie; University of New South Wales<br>Ramke, Jacqueline; LSHTM,<br>Burton, Matthew J; London School of Hygiene and Tropical Medicine<br>Gordon, Iris<br>Zhang, Justine; London School of Hygiene and Tropical Medicine Faculty of Infectious and Tropical Diseases, International Centre for Eye Health, Clinical Research Department<br>Furtado, João; Universidade de São Paulo Faculdade de Medicina de Ribeirão Preto, Division of Ophthalmology<br>Mdala, Shaffi; Queen Elizabeth Central Hospital, Opthalmology Department<br>Kitema, Gatera Fiston; University of Rwanda College of Medicine and Health Sciences, Opthalmology Department<br>Keay, Lisa; University of New South Wales, School of Optometry and Vision Science; The George Institute for Global Health

**Primary Subject Heading**:	Ophthalmology
Secondary Subject Heading:	Public health
Keywords:	PUBLIC HEALTH, OPHTHALMOLOGY, EPIDEMIOLOGY

SCHOLARONE™ Manuscripts

I, the Submitting Author has the right to grant and does grant on behalf of all authors of the Work (as defined in the below author licence), an exclusive licence and/or a non-exclusive licence for contributions from authors who are: i) UK Crown employees; ii) where BMJ has agreed a CC-BY licence shall apply, and/or iii) in accordance with the terms applicable for US Federal Government officers or employees acting as part of their official duties; on a worldwide, perpetual, irrevocable, royalty-free basis to BMJ Publishing Group Ltd ("BMJ") its licensees and where the relevant Journal is co-owned by BMJ to the co-owners of the Journal, to publish the Work in this journal and any other BMJ products and to exploit all rights, as set out in our licence.

The Submitting Author accepts and understands that any supply made under these terms is made by BMJ to the Submitting Author unless you are acting as an employee on behalf of your employer or a postgraduate student of an affiliated institution which is paying any applicable article publishing charge ("APC") for Open Access articles. Where the Submitting Author wishes to make the Work available on an Open Access basis (and intends to pay the relevant APC), the terms of reuse of such Open Access shall be governed by a Creative Commons licence – details of these licences and which Creative Commons licence will apply to this Work are set out in our licence referred to above.

Other than as permitted in any relevant BMJ Author's Self Archiving Policies, I confirm this Work has not been accepted for publication elsewhere, is not being considered for publication elsewhere and does not duplicate material already published. I confirm all authors consent to publication of this Work and authorise the granting of this licence.

For peer review only

Associations between vision impairment and driving and the effectiveness of vision-related interventions: protocol for a systematic review and meta-analysis

Authors: Helen Nguyen[1], Gian Luca Di Tanna[2], Kristy Coxon[3], Julie Brown[2], Kerrie Ren[1], Jacqueline Ramke[4,5], Matthew J Burton[4,6], Iris Gordon[4], Justine H Zhang[4,7], João M. Furtado[8], Shaffi Mdala[9], Gatera Fiston Kitema[10], Lisa Keay[1,2]

Affiliations:

1 School of Optometry and Vision Science, Faculty of Science, The University of New South Wales, NSW, Australia

2 The George Institute for Global Health, Faculty of Medicine, University of New South Wales, NSW, Australia

3 School of Health Sciences, and the Translational Health Research Institute, Western Sydney University, NSW, Australia

4 International Centre for Eye Health, London School of Hygiene & Tropical Medicine, London, United Kingdom, WC1 E7HT

5 School of Optometry and Vision Science, University of Auckland, Auckland, New Zealand

6 Moorfields Eye Hospital, London, UK

7 Manchester Royal Eye Hospital, Manchester, United Kingdom

8 Division of Ophthalmology, Ribeirão Preto Medical School, University of São Paulo, Ribeirão Preto, São Paulo, Brazil

9 Ophthalmology Department, Queen Elizabeth Central Hospital, Blantyre, Malawi

10 Ophthalmology Department, School of Health Sciences, University of Rwanda, Kigali, Rwanda

Corresponding Author: Lisa Keay

Postal Address: School of Optometry and Vision Science, The University of New South Wales, Sydney UNSW NSW 2052, Australia

Email: l.keay@unsw.edu.au

Keywords: visual impairment, road traffic injuries, motor vehicle crashes, driving cessation, driving errors, driving performance, fatal crash involvement

For peer review only - http://bmjopen.bmj.com/site/about/guidelines.xhtml

ABSTRACT

Introduction: Driving is one of the main modes of transport with safe driving requiring a combination of visual, cognitive, and physical skills. With population ageing, the number of people living with vision impairment is set to increase in the decades ahead. Vision impairment may negatively impact an individual's ability to safely drive. The association between vision impairment and motor vehicle crash involvement or driving participation has yet to be systematically investigated. Further, the evidence for the effectiveness of vision-related interventions aimed at decreasing crashes and driving errors has not been synthesised.

Methods and Analysis: A search will be conducted for relevant studies on Medline (Ovid), EMBASE, and Global Health from their inception to March 2020 without date or geographical restrictions. Two investigators will independently screen abstracts and full-texts using Covidence software with conflicts resolved by a third investigator. Data extraction will be conducted on all included studies, and their quality assessed to determine risk of bias using the Joanna Briggs Institute (JBI) Critical Appraisal Tools. Outcome measures include crash risk, driving cessation, and surrogate measures of driving safety (e.g. driving errors and performance). The results of this review will be reported using the Preferred Reporting Items for Systematic Review and Meta-Analysis (PRISMA) guideline. Meta-analysis will be undertaken for outcomes with sufficient data and reported following the Meta-analyses of Observational Studies in Epidemiology (MOOSE) guideline. Where statistical pooling is not feasible or appropriate, narrative summaries will be presented following the Synthesis Without Meta-analysis (SWiM) in systematic reviews guideline.

Ethics and dissemination: This review will only report on published data thus no ethics approval is required. Results will be included in the *Lancet Global Health* Commission on Global Eye Health, published in a peer-reviewed journal, and presented at relevant conferences.

PROSPERO registration number: CRD42020172153

For peer review only

For peer review only - http://bmjopen.bmj.com/site/about/guidelines.xhtml

STRENGTHS AND LIMITATIONS OF THIS STUDY

- Results from this systematic review will present up-to-date evidence for the influence of vision impairment on road traffic injuries (RTIs) and the effectiveness of vision-related interventions.
- As there are no geographic restrictions in the criteria for included studies, this review will capture a large portion of English-language publications in this research area with findings applicable to a global context.
- This review will not restrict the age of the target population allowing evidence on the impact of vision impairment and driving to be documented for all age groups.
- This review only looks at published studies in English, so research from non-English speaking countries will be missed, which could introduce bias
- Another potential limitation is that interventions and outcome measures may be highly heterogeneous which will affect the conclusions drawn from the results and prevent meta-analyses to be conducted for select outcomes.

For peer review only

For peer review only - http://bmjopen.bmj.com/site/about/guidelines.xhtml

INTRODUCTION

According to the World Health Organisation (WHO),(1) approximately 1.35 million people die each year from road traffic injuries (RTIs), making it the eighth leading cause of death globally. Low- and middle income countries (LMICs) have lower rates of vehicle ownership compared to high-income countries (HIC), but over 90% of RTI fatalities occur in LMICs with the highest death rates in Africa. Individuals from lower socioeconomic backgrounds living in HICs are also more likely to be involved in a road crash resulting in injuries. RTIs make up a major proportion of a country's economic and social burden,(2-4) and account for almost 30% of global injury-related disability.(2) In the face of increasing motorisation, achieving absolute reductions in RTIs is a challenge, especially for vulnerable road users such as pedestrians and users of powered two and three wheeler vehicles. This challenge has a direct impact on the UN's Sustainable Development Goals (SDGs), in particularly Target 3.6 which called for a halving of global road deaths by 2020, and Target 11.2 which called for safe and sustainable transport systems, especially for vulnerable road users.(5)

Motor vehicle crashes (MVCs), and by extension RTIs, however, are preventable. Using the Haddon Matrix, an early theory describing the multifactorial nature of RTIs, MVCs are understood to involve host (human), agent (vehicles and equipment), and environmental (physical and socioeconomic) factors(6). This theory has since been used to build the Safe System Approach endorsed in the United Nations Road Safety Collaboration's Decade of Action for Road Safety (2011-2020).(7) In brief, the Safe System Approach aims to prevent MVCs which result in serious injuries or death by addressing four main pillars of focus: 1) safe roads, 2) safe speeds, 3) safe people and 4) safe vehicles.(8) Road safety programs, such as the *Bloomberg Initiative for Global Road Safety (2015-2019)* focus on improving road safety through legislation in LMICs,(9) thus addressing the environmental and agent risks of RTIs. Beyond road infrastructure and vehicle quality, human driving behaviours or 'human factors' also contribute to RTI rates and are an intrinsic part of the Safe System Approach. Safe driving requires individuals to have a range of physical, visual, and cognitive skills. In addition to specific eye diseases, age-related functional declines across a range of domains, including vision, can reduce confidence in driving ability.(10) Poor visual acuity and contrast sensitivity, visual field loss, and glare sensitivity have all been identified as potential factors contributing to poor driving performance and increased MVCs.(11)

Due to the high visual demands needed to drive safely, many countries have set federal and or state-specific standards for vision, mostly for visual acuity. Most countries accept that a visual acuity of at least 6/12 (0.50, 20/40) in the better eye as the requirement for driving. This threshold dictates jurisdictional control used to identify individuals with vision impairment and restrict their access to

For peer review only - http://bmjopen.bmj.com/site/about/guidelines.xhtml

driving privileges. However, a systematic review by Dobbs (2008) suggested that licencing policies aimed at identifying at-risk older drivers may not be effective in decreasing crash rates.(12) This may be because policies which govern licensure and vision screening vary significantly between and within countries. Further, evidence on their effectiveness is inconclusive.(13) Conversely, in-person renewal policies which include vision tests completed at licence renewal centres, have been shown to reduce crash rates in older drivers.(14) An American study analysing data from the National Highway Traffic Safety Administration Fatal Accident Reporting System found drivers aged 70 years and older who underwent visual acuity examinations during their licence renewals had lower fatal crash risks than their non-vision tested peers (RR 0.93; 95% CI 0.89 – 0.97).(15) However, the literature remains divided in its support for using visual acuity alone as a predictor of MVC involvement and high-risk driving behaviours.(16) A Cochrane review, updated twice, examined the benefits of different vision screening procedures, such as visual acuity, visual field (central or peripheral), contrast sensitivity, and useful field of view (UFOV) tests, in randomised-controlled trials (RCTs) aimed at preventing RTIs and fatalities in older drivers.(13, 17) Unfortunately, no RCTs met the inclusion criteria for the review at the time these reviews were conducted.

There is substantial literature investigating how vision impairment, and other aspects of function, affect road safety. Measures of driving safety have included indirect measures such as performance on driving simulators, on-road driving assessments, naturalistic driving or in-vehicle monitoring as well as direct measures of RTI and MVC rates from self-report or administrative datasets.(18, 19) However, the evidence for the influence of vision loss on MVCs and the corresponding benefits of interventions to restore vision have not been systematically evaluated. Since older drivers have higher crash involvement(20) and greater prevalence of eye diseases due to natural age-related declines in vision,(21) most research investigate older drivers and their risks of crashes and injuries. However, it is important to document the impact of vision impairment across all age groups. Further, information is also needed about specific eye diseases and types of vision impairment to inform interventions to screen for poor vision in drivers, and interventions to rehabilitate vision, thereby enhancing driver safety and continued ability to drive. This is especially important for older adults who rely on driving to remain independent and connected with their community. The loss of the ability to drive and the eventual retirement from driving has been linked to higher symptoms of depression and poorer health in older adults.(22)

The aim of this systematic review is to: 1) describe the associations between vision impairment and risk of road crash involvement across the lifespan, and 2) evaluate vision-related interventions to reduce crash risk. Secondary outcomes are driving cessation and surrogate measures of crash risk such as on-road driving errors.

For peer review only - http://bmjopen.bmj.com/site/about/guidelines.xhtml

METHODS AND ANALYSIS

This systematic review protocol was drafted using the International Prospective Register of Systematic Reviews (PROSPERO) as a guideline and registered in PROSPERO (28/04/2020; CRD42020172153 [https://www.crd.york.ac.uk/prospero/display_record.php?ID=CRD42020172153]). Any changes to the protocol will be updated in PROSPERO. The protocol is prepared in accordance to the Preferred Reporting Items for Systematic review and Meta-Analysis Protocols (PRISMA-P) statement (Appendix no. 1).(23)

Eligibility Criteria

This review will include human studies in the English language with full-text available. Unlike the two previous Cochrane reviews which only included RCTs, this review will consider both interventional (RCTs and quasi-experimental) and observational (cohort, cross-sectional, and case-control) studies. Systematic reviews will be included if meta-analysis was performed. For systematic reviews without meta-analysis, the reference list will be examined for potentially relevant articles, but the systematic review itself will not be included. All literature reviews, commentary articles, dissertations, abstracts, editorials, and conference presentations will be excluded.

All studies must report on at least one of the outcome variables, described in the following section, which include MVC involvement and surrogate measures of driving safety such as driving errors and performance scores and driving cessation. Studies investigating either self-regulatory behaviours, such as night driving avoidance and decreasing travel mileage, or self-reported measures of driving safety, will be excluded. To obtain data on driving scores and performance, studies using on-road driving tests, which include closed-circuit routes and those combining both closed and real-road driving tracks, and naturalistic driving with in-vehicle monitoring will be included. Even though closed-circuits may not reflect true on-road driving conditions, tests for common driving maneuverers such as road signage recognition, hazard recognition and avoidance, reversing, and gap perception are able to be recreated on these routes.(24) Driving errors and driving performance scores on the on-road driving tests can come from fitted in-vehicle monitoring technologies or trained observers. To restrict the scope of the study to direct measures of driving, studies which used driving simulators will be excluded as these are laboratory studies with only indirect measures of driving performance. MVC involvement cannot be measured and real-life driving experiences, such as limited exposure to driving at night, in bad weather, or during rush hour, may not be reflected in a simulation.(25) Additionally, the validity of driving simulator results are highly dependent upon the type of simulation program used and what kind of driving manoeuvre is being

For peer review only - http://bmjopen.bmj.com/site/about/guidelines.xhtml

investigated.(26) As this review is interested in the MVC involvement and driving abilities of individuals who drive and their habitual vision, studies which simulate impairments in vision will also be excluded.

The population of focus will be drivers of four-wheeled motorised vehicles such as cars, buses, and trucks. Unlike the two Cochrane reviews mentioned above which only focused on older drivers, this review will include drivers of all ages. Studies of drivers who have specific medical conditions (e.g. dementia, epilepsy, stroke, and history of medical events such as syncope), or vision difficulties due to other medical factors (e.g. hemianopia caused by brain damage) will not be included. Similarly, articles where vision status is not reported will be excluded.

Exposures in the included studies will encompass all types of vision impairment including visual acuity, contrast sensitivity, visual field loss as well as impairments associated with specific eye diseases including but not limited to glaucoma, cataracts, aged-related macular degeneration (AMD), diabetic retinopathy (DR), stereopsis disorders, and colour vision deficiencies. Vision impairments can be categorised by the specific eye diseases or by specific measures of vision which can negatively impact normal everyday functioning. Even though it is not necessary for all included studies to report on vision-related interventions, studies which do report on interventions can include procedures such as vision screening, refractive correction, cataract surgery or other measures to restore and improve vision of drivers in order to maintain driving participation, promote safe driving and reduce risk of crash involvement. The exposure comparators of included studies will be drivers who either do not have a vision impairment or have not received a vision-related intervention, within a timeframe chosen by the study in question.

Outcome Measures

The primary outcome measure is MVC involvement including fatal MVC involvement. Data on crash involvement and its severity can either come from self-reported surveys or data linkage with government and/or hospital records. Data from self-reported surveys will ensure that MVCs which were not serious enough to warrant a police or hospital report will be also be included.

Driving cessation and surrogate measures of driving safety will be the secondary outcomes. The surrogate measures of driving safety can include scores of driving performance from on-road driving tests or 'naturalistic' in-vehicle monitoring looking at manoeuvres such as lane keeping, braking, and abidance of road signage like traffic lights, stop and give way signs. To account for differences in the criteria used by trained observers to evaluate the driving performance scores on on-road driving tests, a pass/fail threshold for driving performance scores specific to this review will be decided upon by all investigators in order to synthesise results.

For peer review only - http://bmjopen.bmj.com/site/about/guidelines.xhtml

Search Strategy

Electronic database search will be conducted by the Cochrane Eyes and Vision Information Specialist (IG) on Medline (Ovid), EMBASE, and Global Health from their inception to March 2020. Appendices 2, 3, and 4 shows the search strategies for Medline, EMBASE, and Global Health, respectively. Additional potentially relevant studies will be sought by experts in the field by checking the reference lists and citations of included studies, and checking the reference list of narrative systematic reviews identified in the search.

Data Collection and Analysis

Data Management and Selection

Each title and abstract will be screened by two investigators independently (from HN, KR, JR, JZ, SM, JF, GFK) using Covidence systematic review management software (Veritas Health Innovation, Melbourne, Australia; available at https://www.covidence.org/home). Full-text review of potentially relevant articles will then be conducted by two investigators independently. Discrepancies will be discussed and resolved via consultation with a third investigator.

Data Extraction

Data extraction will be completed independently by two investigators (from among the same seven investigators). Data from included studies will be extracted using adaptions of the Joanna Briggs Institute (JBI) template for systematic reviews and observational studies (including cohort, cross-sectional, and case-control studies).(27) Adapted Cochrane templates will be used to extract data from randomized controlled trials and quasi-experimental studies.(28)

Quality Assessment

A quality assessment to determine an overall risk of bias will be carried out on all included studies independently by two investigators (from the seven investigators mentioned previously). Conflicts will be resolved by a third investigator. Relevant JBI critical appraisal tools will be used to evaluate randomised controlled trials, quasi-experimental studies, systematic reviews, cohort studies, cross-sectional studies, and case-control studies.(29)

Data Synthesis Strategy

Measures of association between vision impairment/vision related interventions and MVC involvement, driving cessation and surrogate measures of driving safety will be summarized according to the outcome measures reported in the primary studies. In particular, appropriate hazard ratio (HR), risk ratio (RR), and odds ratio (OR) for binary data and (standardized) mean

For peer review only - http://bmjopen.bmj.com/site/about/guidelines.xhtml

differences for continuous data will be statistically pooled. When the same outcome is reported as dichotomous data in some studies and as continuous data in others, these studies will be pooled by expressing the ORs as standardized mean differences and vice versa.(30) P-values of the driving outcomes will also be reported where appropriate.

Where it is not possible or suitable to statistically pool the studies, a narrative summary of the findings will be used instead. Narrative summaries will follow the Synthesis Without Meta-analysis (SWiM) reporting guidelines.(31) Heterogeneity across all included studies with sufficient data will be assessed clinically, methodologically and statistically. Clinical heterogeneity will be accessed by comparing the differences between the participant characteristics (e.g. age, sex, eye disease, driving mileage, licence status or other available measures of driving exposure), interventions and outcomes measured. The design and quality of included studies will be compared to access methodological heterogeneity. Statistical heterogeneity across studies will be explored by formal statistical test of heterogeneity, subgroup analyses and, if feasible, by meta-regression. Inconsistency of the effect sizes across the studies will be assessed by the proportion of variability in the effect sizes of the included studies due to heterogeneity (and not by sampling error) using I^2. Estimates will be pooled using random effects models with fixed effect models results also reported regardless of the values of I^2, and prediction intervals to allow for expected effects of future studies to be extrapolated based upon the current evidence.(32)

The following outcomes will be assessed using meta-analysis where feasible according to data availability: crash involvement, driving cessation, and surrogate measures of un-safe driving i.e. driving errors and driving performance. Furthermore meta-analyses for each of the different eye diseases, and studies from LMIC settings will also be performed independently for each outcome of interest if possible. As there is no age restriction on the focus population, results on age will be synthesised by assessing specific subgroup analysis and/or meta-regression which may partially explain heterogeneity across studies in the pooled effect size. The Meta-analysis of Observational Studies in Epidemiology (MOOSE) guidelines will be used to guide reporting.(33) The Grading of Recommendations Assessment, Development and Evaluation (GRADE) approach will be used to assess the quality of evidence in the meta-analyses.(34)

Sensitivity analysis will be performed on low risk of bias studies whilst the meta-analysis will include all studies. This will assist with verifying the strength of the study findings and to assess how different methodologies, sample sizes and statistical analyses have affected this study's results. Furthermore, funnel plots will be used to assess publication bias.

For peer review only - http://bmjopen.bmj.com/site/about/guidelines.xhtml

Corresponding authors from publications dated 2010 onwards with missing data of potential use will be approached via email, up to a maximum of 3 attempts, to request further information. Any unobtainable data will be noted alongside all attempts to contact the respective authors. Even though only available data will be used for the meta-analysis, the effects of any missing data will be considered and their effects discussed in the overall final review.

Patient and Public Involvement

This review will only be looking at existing published literature. No patient or public involvement is currently planned for the design and execution of this review, however public participation may be sought for this review's dissemination.

ETHICS AND DISSEMINATION

As this review will only be focusing on currently published literature, ethics approval is not required. Results from this systematic review will be published in an open peer-reviewed journal and will form part of the ongoing *Lancet Global Health* Commission on Global Eye Health.(35) Where relevant, it will also be presented at conferences.

DISCUSSION

Significance of this review

The findings of this systematic review may influence future road safety and licencing policies on driving for drivers with vision impairment. By understanding the visual factors contributing to MVCs, vision-related screening tests for licencing may be reconsidered and updated to increase relevance to driving safety. As mentioned previously, most reviews on driving with vision impairment have been limited to older drivers and the effects of different licencing renewal procedures on their ability to drive. Even though older drivers are at higher risk,(21) this review will seek to capture data on driving and vision impairment for all age groups.

The eligibility criteria for included studies for this review will ensure that global data on vision and driving will be captured. Currently, MVC-related societal burdens and injury- related disability burdens in LMICs are poorly understood which may partially explain why cost-effective interventions in these countries are rarely undertaken.(2) LMICs tend to focus on legislative interventions, followed by education/training workshops, public awareness campaigns, enforcement measures, speed control and infrastructure improvements.(36) Current data on human factors specifically related to vision impairment in LMICs reported in this review may inform future evidence-based policies on licencing and/or screening policies to address these gaps.

For peer review only - http://bmjopen.bmj.com/site/about/guidelines.xhtml

For peer review only

Results from this review may also provide additional evidence on the impact of eye-disease specific interventions on quality of life factors, especially those related to driving and the ability to drive. Interventions to improve and optimise vision are needed for drivers, in recognition of the importance of continued safe driving. This greater awareness in turn will also provide evidence for policies around road safety for individuals with vision impairments.

For peer review only

For peer review only - http://bmjopen.bmj.com/site/about/guidelines.xhtml

REFERENCES

1 World Health Organisation. Road Traffic Injuries 2020; https://www.who.int/news-room/fact-sheets/detail/road-traffic-injuries (accessed April 2020)

2 Bachani AM, Peden M, Gururaj G, et al. Road Traffic Injuries. In: Mock CN, Nugent R, Kobusingye O, Smith KR, eds. Injury Prevention and Environmental Health. Washington (DC): The International Bank for Reconstruction and Development / The World Bank © 2017 International Bank for Reconstruction and Development / The World Bank.; 2017.

3 Chen S, Kuhn M, Prettner K, et al. The global macroeconomic burden of road injuries: estimates and projections for 166 countries. *Lancet Planet Health* 2019;3(9):e390-e8. doi: 10.1016/S2542-5196(19)30170-6

4 Wijnen W, Stipdonk H. Social costs of road crashes: An international analysis. *Accid Anal Prev* 2016;94:97-106. doi: 10.1016/j.aap.2016.05.005 [published Online First: 3 June 2016]

5 United Nations. #Envision2030: 17 goals to transform the world for persons with disabilities; https://www.un.org/development/desa/disabilities/envision2030.html (accessed April 2020)

6 Haddon W, Jr. Energy damage and the ten countermeasure strategies. *J Trauma* 1973;13(4):321-31. doi: 10.1177/001872087301500407

7 The Department of Infrastructure, Regional Development and Communications: Australian Government. Decade of Action for Road Safety 2011-2020 2014; https://www.infrastructure.gov.au/roads/safety/decade_of_action/index.aspx (accessed April 2020)

8 Towards Zero Foundation. The Safe System; http://www.towardszerofoundation.org/thesafesystem/ (accessed 30 July 2020)

9 Global Road Safety Partnership. Bloomberg Initiative for Global Road Safety; https://www.grsproadsafety.org/programmes/bloomberg-initiative-global-road-safety/ (accessed April 2020)

10 Miller SM, Taylor-Piliae RE, Insel KC. The association of physical activity, cognitive processes and automobile driving ability in older adults: A review of the literature. *Geriatr Nurs* 2016;37(4):313-20. doi: 10.1016/j.gerinurse.2016.05.004 [published Online First: 31 May 2016]

11 Rubin GS, Ng ES, Bandeen-Roche K, et al. A prospective, population-based study of the role of visual impairment in motor vehicle crashes among older drivers: the SEE study. *Invest Ophthalmol Vis Sci* 2007;48(4):1483-91. doi: 10.1167/iovs.06-0474

12 Dobbs BM. Aging baby boomers--a blessing or challenge for driver licensing authorities. *Traffic Inj Prev* 2008;9(4):379-86. doi: 10.1080/15389580802045823

13 Desapriya E, Harjee R, Brubacher J, et al. Vision screening of older drivers for preventing road traffic injuries and fatalities. *Cochrane Database Syst Rev* 2014(2):Cd006252. doi: 10.1002/14651858.CD006252.pub3

For peer review only - http://bmjopen.bmj.com/site/about/guidelines.xhtml

14 Grabowski DC, Campbell CM, Morrisey MA. Elderly licensure laws and motor vehicle fatalities. *JAMA* 2004;291(23):2840-6. doi: 10.1001/jama.291.23.2840

15 Levy DT, Vernick JS, Howard KA. Relationship between driver's license renewal policies and fatal crashes involving drivers 70 years or older. *JAMA* 1995;274(13):1026-30. doi: 0.1001/jama.1995.03530130032024

16 Owsley C, McGwin G, Jr. Vision impairment and driving. *Surv Ophthalmol* 1999;43(6):535-50.

17 Subzwari S, Desapriya E, Babul-Wellar S, et al. Vision screening of older drivers for preventing road traffic injuries and fatalities. *Cochrane Database Syst Rev* 2009(1):Cd006252. doi: 10.1002/14651858.CD006252.pub2

18 Imprialou M, Quddus M. Crash data quality for road safety research: Current state and future directions. *Accid Anal Prev* 2019;130:84-90. doi: 10.1016/j.aap.2017.02.022

19 Kaye SA, Lewis I, Freeman J. Comparison of self-report and objective measures of driving behavior and road safety: A systematic review. *J Safety Res* 2018;65:141-51. doi: 10.1016/j.jsr.2018.02.012

20 Owsley C, Ball K, Sloane ME, et al. Visual/cognitive correlates of vehicle accidents in older drivers. *Psychol Aging* 1991;6(3):403-15. doi: 10.1037//0882-7974.6.3.403

21 Wood JM, Black AA. Ocular disease and driving. *Clin Exp Optom*. 2016;99(5):395-401. doi: 10.1111/cxo.12391 [published Online First: 7 May 2016]

22 Ragland DR, Satariano WA, MacLeod KE. Driving cessation and increased depressive symptoms. *J Gerontol A Biol Sci Med Sci* 2005;60(3):399-403. doi: 10.1093/gerona/60.3.399

23 Shamseer L, Moher D, Clarke M, et al. Preferred reporting items for systematic review and meta-analysis protocols (PRISMA-P) 2015: elaboration and explanation. *BMJ* 2015;350:g7647. doi: 10.1136/bmj.g7647

24 Wood JM. Age and visual impairment decrease driving performance as measured on a closed-road circuit. *Hum factors*. 2002;44(3):482-94. doi: 10.1518/0018720024497664

25 Keay L, West SK. Vision and Driving Performance in Elderly. In: Scholl HPN, Massof RW, West SK eds. Ophthalmology and the Ageing Society. Berlin Heidelberg: Springer-Verlag 2013:193-207.

26 Shechtman O, Classen S, Awadzi K, et al. Comparison of driving errors between on-the-road and simulated driving assessment: a validation study. *Traffic Inj Prev* 2009;10(4):379-85. doi: 10.1080/15389580902894989

27 The Joanna Briggs Institute. Joanna Briggs Institute Reviewer's Manual. 2017. Available from: https://reviewersmanual.joannabriggs.org/ (accessed April 2020)

28 Cochrane: Developmental PaLP. Data Extraction Forms 2014 [Available from: https://dplp.cochrane.org/data-extraction-forms (accessed April 2020).

29 University of Adelaide. Critical Appraisal Tools; https://joannabriggs.org/ebp/critical_appraisal_tools (assessed April 2020)

For peer review only - http://bmjopen.bmj.com/site/about/guidelines.xhtml

30 Anzures-Cabrera J, Sarpatwari A, Higgins JP. Expressing findings from meta-analyses of continuous outcomes in terms of risks. *Stat Med* 2011;30(25):2967-85. doi: 10.1002/sim.4298 [published Online First: 8 August 2011]

31 Campbell M, McKenzie JE, Sowden A, et al. Synthesis without meta-analysis (SWiM) in systematic reviews: reporting guideline. *BMJ* 2020;368:l6890. doi: 10.1136/bmj.l6890

32 IntHout J, Ioannidis JP, Rovers MM, et al. Plea for routinely presenting prediction intervals in meta-analysis. *BMJ Open* 2016;6(7):e010247. doi: 10.1136/bmjopen-2015-010247

33 Stroup DF, Berlin JA, Morton SC, et al. Meta-analysis of observational studies in epidemiology: a proposal for reporting. Meta-analysis Of Observational Studies in Epidemiology (MOOSE) group. *JAMA* 2000;283(15):2008-12. doi: 10.1001/jama.283.15.2008

34 Iorio A, Spencer FA, Falavigna M, et al. Use of GRADE for assessment of evidence about prognosis: rating confidence in estimates of event rates in broad categories of patients. *BMJ* 2015;350:h870. doi: 10.1136/bmj.h870

35 Burton MJ, Faal HB, Ramke J, et al. Announcing The Lancet Global Health Commission on Global Eye Health. *Lancet Glob Health* 2019;7(12):e1612-e3. doi: 10.1016/S2214-109X(19)30450-4 [published Online First: 9 October 2019]

36 Staton C, Vissoci J, Gong E, et al. Road Traffic Injury Prevention Initiatives: A Systematic Review and Metasummary of Effectiveness in Low and Middle Income Countries. *PloS One* 2016;11(1):e0144971. doi: 10.1371/journal.pone.0144971

For peer review only

For peer review only - http://bmjopen.bmj.com/site/about/guidelines.xhtml

Authors' Contributions: HN, LK, and JR conceived the idea for the review. HN drafted and revised the protocol with suggestions from LK, JR, JB, KC, GLdT, KR, MJB, JZ, IG, JFM, SM, and GFK who reviewed the protocol and provided feedback on the draft. IG constructed the search. The final version of the manuscript was approved by all authors.

Funding Statement: MJB is supported by the Wellcome Trust (207472/Z/17/Z). JR is a Commonwealth Rutherford Fellow, funded by the UK government through the Commonwealth Scholarship Commission in the UK. The Lancet Global Health Commission on Global Eye Health is supported by The Queen Elizabeth Diamond Jubilee Trust, The Wellcome Trust, Sightsavers, The Fred Hollows Foundation, The SEVA Foundation, The British Council for the Prevention of Blindness and Christian Blind Mission.

Acknowledgements: None.

Competing Interests: None declared.

Number of Tables: 0

Number of Figures: 0

Data Sharing Statement: Data generated from this review will be available upon reasonable request from HN.

Word Count: 3376

For peer review only

Appendix 1. Preferred Reporting Items for Systematic review and Meta-analysis Protocols (PRISMA-P) 2015 Checklist

Section and topic	Item No	Checklist item	Page Number
**ADMINISTRATIVE INFORMATION**			
Title:			
Identification	1a	Identify the report as a protocol of a systematic review	1
Update	1b	If the protocol is for an update of a previous systematic review, identify as such	N/A
Registration	2	If registered, provide the name of the registry (such as PROSPERO) and registration number	2, 6
Authors:			
Contact	3a	Provide name, institutional affiliation, e-mail address of all protocol authors; provide physical mailing address of corresponding author	1
Contributions	3b	Describe contributions of protocol authors and identify the guarantor of the review	8, 15
Amendments	4	If the protocol represents an amendment of a previously completed or published protocol, identify as such and list changes; otherwise, state plan for documenting important protocol amendments	N/A
Support:			
Sources	5a	Indicate sources of financial or other support for the review	15
Sponsor	5b	Provide name for the review funder and/or sponsor	15
Role of sponsor or funder	5c	Describe roles of funder(s), sponsor(s), and/or institution(s), if any, in developing the protocol	15
**INTRODUCTION**			
Rationale	6	Describe the rationale for the review in the context of what is already known	4, 5
Objectives	7	Provide an explicit statement of the question(s) the review will address with reference to participants, interventions, comparators, and outcomes (PICO)	5
**METHODS**			
Eligibility criteria	8	Specify the study characteristics (such as PICO, study design, setting, time frame) and report characteristics (such as years considered, language, publication status) to be used as criteria for eligibility for the review	6, 7
Information sources	9	Describe all intended information sources (such as electronic databases, contact with study authors, trial registers or other grey literature sources) with planned dates of coverage	6, 8

For peer review only

Search strategy	10	Present draft of search strategy to be used for at least one electronic database, including planned limits, such that it could be repeated	Appendix no. 2-4
Study records:			
Data management	11a	Describe the mechanism(s) that will be used to manage records and data throughout the review	8
Selection process	11b	State the process that will be used for selecting studies (such as two independent reviewers) through each phase of the review (that is, screening, eligibility and inclusion in meta-analysis)	8
Data collection process	11c	Describe planned method of extracting data from reports (such as piloting forms, done independently, in duplicate), any processes for obtaining and confirming data from investigators	8
Data items	12	List and define all variables for which data will be sought (such as PICO items, funding sources), any pre-planned data assumptions and simplifications	6, 7, 8
Outcomes and prioritization	13	List and define all outcomes for which data will be sought, including prioritization of main and additional outcomes, with rationale	7
Risk of bias in individual studies	14	Describe anticipated methods for assessing risk of bias of individual studies, including whether this will be done at the outcome or study level, or both; state how this information will be used in data synthesis	8, 9
Data synthesis	15a	Describe criteria under which study data will be quantitatively synthesised	8, 9
	15b	If data are appropriate for quantitative synthesis, describe planned summary measures, methods of handling data and methods of combining data from studies, including any planned exploration of consistency (such as I^2, Kendall's τ)	8, 9
	15c	Describe any proposed additional analyses (such as sensitivity or subgroup analyses, meta-regression)	8, 9
	15d	If quantitative synthesis is not appropriate, describe the type of summary planned	2, 9
Meta-bias(es)	16	Specify any planned assessment of meta-bias(es) (such as publication bias across studies, selective reporting within studies)	8, 9
Confidence in cumulative evidence	17	Describe how the strength of the body of evidence will be assessed (such as GRADE)	9

For peer review only

Appendix 2. MEDLINE (OVID) search strategy

1. exp Eye Diseases/
2. exp Cataract Extraction/
3. Lens Implantation, Intraocular/
4. Lenses, Intraocular/
5. cataract$.tw.
6. ((intraocular or intra ocular) adj3 lens$).tw.
7. (IOL or IOLs).tw.
8. Vision Tests/
9. Visual Acuity/
10. exp Refractive Errors/
11. Visual Fields/
12. Visual Field Tests/
13. Contrast Sensitivity/
14. Depth Perception/
15. (visual adj2 (acuit$ or field$)).tw.
16. contrast sensitivity.tw.
17. (depth perception or stereopsis).tw.
18. ((impair$ or decreas$ or declin$) adj3 (vision or visual$ or sight$)).tw.
19. (improv$ adj3 (vision or visual$ or sight$)).tw.
20. ((visual or vision) adj2 function$).tw.
21. exp Vision, Ocular/
22. Vision Screening/
23. or/1-22
24. Mass Screening/
25. ((eye$ or sight or vision or visual$) adj2 (test$ or screen$ or exam$ or diagnos$ or assess$)).tw
26. 24 and 25
27. 23 or 26
28. exp Motor Vehicles/
29. exp Automobile Driving/
30. Accidents, Traffic/
31. (driver$ or driving).tw.
32. (automobile$ or car or cars or vehicle$).tw.
33. (motoring or motorcar or "motor car" or "motor cars").tw.
34. crash$.tw.
35. ((road or traffic) adj2 injur$).tw.
36. ((road or traffic or motor) adj2 (accident$ or incident$)).tw.
37. ((road or traffic or motor) adj2 collision$).tw.
38. or/28-37
39. epidemiologic studies/ or case-control studies/ or cohort studies/ or observational study/ or follow-up studies/ or longitudinal studies/ or prospective studies/ or retrospective studies/ or controlled before-after studies/ or cross-sectional studies/ or historically controlled study/ or interrupted time series analysis/
40. epidemiologic methods/ or focus groups/ or interviews as topic/ or exp "surveys and questionnaires"/
41. epidemiologic research design/ or control groups/ or cross-over studies/ or double-blind method/ or meta-analysis as topic/ or network meta-analysis/ or random allocation/ or single-blind method/
42. epidemiologic methods/ or clinical trials as topic/ or feasibility studies/ or multicenter studies as topic/ or pilot projects/ or sampling studies/ or twin studies as topic/

For peer review only

43. randomized controlled trial/ or controlled clinical trials as topic/ or randomized controlled trials as topic/
44. comparative study/ or evaluation studies/ or meta-analysis/ or review/ or multicenter study/ or "systematic review"/ or validation studies/
45. health surveys/
46. outcome assessment, health care/
47. risk factors/
48. self report/
49. (population or cohort or observation$ or intervention$ or prospective or retrospective or comparative).tw.
50. (questionnaire$ or survey$).tw.
51. (randomized or randomised or randomly or RCT).tw.
52. (systematic review or meta-analysis).tw.
53. (before adj2 after).tw.
54. (case$ adj2 control$).tw.
55. (cross adj1 section$).tw.
56. or/39-55
57. 27 and 38
58. 56 and 57
59. vehicle-controlled.tw.
60. (vehicle adj3 inject$).tw.
61. 59 or 60
62. 58 not 61
63. (animal$ or mouse or mice$ or dog or canine or rat or rats or primate$).ti.
64. (dry eye or cell$ or mutation$ or genes or genome or sequencing).ti.
65. or/63-64
66. 62 not 65
67. limit 66 to english language
68. exp case reports/
69. (case adj2 report$).tw.
70. 68 or 69
71. 67 not 70
72. limit 71 to (editorial or letter)
73. 71 not 72

For peer review only

Appendix 3. EMBASE Search Strategy

1. exp eye disease/
2. exp cataract extraction/
3. lens implantation/
4. lens implant/
5. cataract$.tw.
6. ((intraocular or intra ocular) adj3 lens$).tw.
7. (IOL or IOLs).tw.
8. vision test/
9. visual acuity/
10. refractive error/
11. visual field/
12. perimetry/
13. contrast sensitivity/
14. depth perception/
15. (visual adj2 (acuit$ or field$)).tw.
16. contrast sensitivity.tw.
17. (depth perception or stereopsis).tw.
18. ((impair$ or decreas$ or declin$) adj3 (vision or visual$ or sight$)).tw.
19. (improv$ adj3 (vision or visual$ or sight$)).tw.
20. ((visual or vision) adj2 function$).tw.
21. vision/
22. or/1-21
23. mass screening/
24. ((eye$ or sight or vision or visual$) adj2 (test$ or screen$ or exam$ or diagnos$ or assess$)).tw.
25. 23 and 24
26. 22 or 25
27. exp car driving/
28. exp motor vehicle/
29. traffic accident/
30. (driver$ or driving).tw.
31. (automobile$ or car or cars or vehicle$).tw.
32. (motoring or motorcar or "motor car" or "motor cars").tw.
33. crash$.tw.
34. ((road or traffic) adj2 injur$).tw.
35. ((road or traffic or motor) adj2 (accident$ or incident$)).tw.
36. ((road or traffic or motor) adj2 collision$).tw.
37. or/27-36
38. study design/
39. controlled clinical trial/
40. case control study/
41. cohort analysis/
42. observational study/
43. follow up/
44. longitudinal study/
45. prospective study/
46. retrospective study/
47. epidemiology/
48. cross-sectional study/
49. control group/

For peer review only

50. crossover procedure/
51. "meta analysis (topic)"/
52. network meta-analysis/
53. randomization/
54. single blind procedure/
55. double blind procedure/
56. "clinical trial (topic)"/
57. "controlled clinical trial (topic)"/
58. "randomized controlled trial (topic)"/
59. "multicenter study (topic)"/
60. feasibility study/
61. pilot study/
62. comparative study/
63. evaluation study/
64. multicenter study/
65. randomized controlled trial/
66. meta analysis/
67. "systematic review"/
68. validation study/
69. interview/
70. questionnaire/
71. outcome assessment/
72. "systematic review (topic)"/
73. health survey/
74. risk factor/
75. self report/
76. evidence based practice/
77. (population or cohort or observation$ or intervention$ or prospective or retrospective or comparative).tw.
78. (questionnaire$ or survey$).tw.
79. (randomized or randomised or randomly or RCT).tw.
80. (systematic review or meta-analysis).tw.
81. (before adj2 after).tw.
82. (case$ adj2 control$).tw.
83. (cross adj1 section$).tw.
84. or/38-83
85. 26 and 37
86. 84 and 85
87. vehicle-controlled.tw.
88. (vehicle adj3 inject$).tw.
89. or/87-88
90. 86 not 89
91. (animal$ or mouse or mice$ or dog or canine or rat or rats or primate$).ti.
92. (dry eye or cell$ or mutation$ or genes or genome or sequencing).ti.
93. or/91-92
94. 90 not 93
95. limit 94 to conference abstract status
96. 94 not 95
97. limit 96 to english language
98. exp case report/
99. (case adj2 report$).tw.

For peer review only

100. or/98-99
101. 97 not 100
102. limit 101 to (conference paper or "conference review" or editorial or letter or note)
103. 101 not 102

For peer review only

Appendix 4. GLOBAL HEALTH Search Strategy

1. exp eye diseases/
2. exp vision disorders/
3. cataract$.tw.
4. ((intraocular or intra ocular) adj3 lens$).tw.
5. (IOL or IOLs).tw.
6. (visual adj2 (acuit$ or field$)).tw.
7. contrast sensitivity.tw.
8. (depth perception or stereopsis).tw.
9. ((impair$ or decreas$ or declin$) adj3 (vision or visual$ or sight$)).tw.
10. (improv$ adj3 (vision or visual$ or sight$)).tw.
11. ((visual or vision) adj2 function$).tw.
12. ((eye$ or sight or vision or visual$) adj2 (test$ or screen$ or exam$ or diagnos$ or assess$)).tw.
13. or/1-12
14. drivers/
15. vehicles/
16. motor cars/
17. traffic/
18. traffic accidents/
19. (driver$ or driving).tw.
20. (automobile$ or car or cars or vehicle$).tw.
21. (motoring or motorcar or "motor car" or "motor cars").tw.
22. crash$.tw.
23. ((road or traffic) adj2 injur$).tw.
24. ((road or traffic or motor) adj2 (accident$ or incident$)).tw.
25. ((road or traffic or motor) adj2 collision$).tw.
26. or/14-25
27. cohort studies/
28. case-control studies/
29. longitudinal studies/
30. retrospective studies/
31. epidemiology/
32. exp clinical trials/
33. randomized controlled trials/
34. feasibility studies/
35. pilot projects/
36. meta-analysis/
37. systematic reviews/
38. reviews/
39. questionnaires/
40. surveys/
41. epidemiological surveys/
42. risk factors/
43. (population or cohort or observation$ or intervention$ or prospective or retrospective or comparative).tw.
44. (questionnaire$ or survey$).tw.
45. (randomized or randomised or randomly or RCT).tw.
46. (systematic review or meta-analysis).tw.
47. (before adj2 after).tw.
48. (case$ adj2 control$).tw.

For peer review only

49. (cross adj1 section$).tw.
50. or/27-49
51. 13 and 26
52. 50 and 51
53. (animal$ or mouse or mice$ or dog or canine or rat or rats or primate$).ti.
54. (dry eye or cell$ or mutation$ or genes or genome or sequencing).ti.
55. 53 or 54
56. 52 not 55
57. limit 56 to english language
58. case reports/
59. (case adj2 report$).tw.
60. 58 or 59
61. 57 not 60
62. limit 61 to (conference or conference paper or conference proceedings or correspondence or editorial or thesis)
63. 61 not 62

For peer review only
